# Perovskite nanocrystals-in-glass hierarchical structures enable stable continuous-wave random lasers

**DOI:** 10.1126/sciadv.adz8460

**Published:** 2026-01-16

**Authors:** Xinkuo Li, Chenduan Chen, Ke Sun, Linhan Li, Zhu Xiao, Zhou Li, Yuanzheng Yue, Jianrong Qiu, Dezhi Tan

**Affiliations:** ^1^ State Key Laboratory of Extreme Photonics and Instrumentation, College of Optical Science and Engineering, College of Optical Science and Engineering, and School of Materials Science and Engineering, Zhejiang University, Hangzhou 310027, China.; ^2^ China International Science & Technology Cooperation Base for Laser Processing Robotics, Wenzhou University, Wenzhou 325035, China.; ^3^ School of Materials Science and Engineering, Central South University, Changsha 410083, China.; ^4^ Department of Chemistry and Bioscience, Aalborg University, 9220 Aalborg, Denmark.; ^5^ Zhejiang Lab, Hangzhou 311121, China.

## Abstract

Encapsulating perovskite nanocrystals (PNCs) in glass enables enhanced stability of PNCs and numerous applications such as random lasers. However, preparing PNCs and tuning their properties in glass is energy consuming because of high processing temperature and long processing time, and continuous-wave (CW) random lasers have not been achieved. Here, we report modulation of the structure, photoluminescence, and lasing properties of PNCs in glass at temperatures well below the glass transition temperature with a short processing period. We generate tunable PNCs in glass via nanophase separation and ion exchange in the perovskite domains. PNCs-in-glass hierarchical structures are created by controlling nanophase separation and crystallization of PNCs. Substantially increased scattering in the hierarchical structures enables stable CW single-mode random lasing with an ultralow threshold of 52.6 milliwatts per square centimeter. We achieve flexible CW random lasers by incorporating hierarchical structures into the polydimethylsiloxane film. The random lasers are used in speckle-free laser imaging and dynamic holographic displays.

## Teaser

Perovskite nanocrystals-in-glass hierarchical structures achieve stable continuous-wave random lasing.

## INTRODUCTION

Perovskite nanocrystals (PNCs) have received notable attention due to their exceptional optoelectronic properties, including a widely tunable bandgap, remarkable defect tolerance, large absorption efficiency, and high quantum yield (QY) (*1*, *2*). These attributes make PNCs highly promising for a range of advanced applications such as light-emitting devices (*1*), sensors (*3*), photovoltaic devices (*4*), and lasers (*2*). However, PNCs suffer from poor chemical durability due to their intrinsic ionic nature. To address this limitation, passivation and encapsulation are required for PNCs and their devices (*5*, *6*). Encouragingly, glass has been demonstrated to be an excellent matrix for protecting PNCs, offering enhanced stability and preserving their superior optoelectronic properties (*7*–*9*). Unfortunately, preparing and optimizing NC-in-glass composites (NGCs) commonly rely on the solid-phase separation from the glass matrix through heat treatment (*9*, *10*). High temperatures that typically exceed the glass transition temperature (*T*_g_), and long heating times are necessary to realize precipitation of NCs in oxide glasses and to tune their optical properties. This is because an increase in temperatures above *T*_g_ can lower the viscosity of oxide glasses, thereby increasing ion diffusion for NC nucleation (*11*, *12*).

Glass can be an important optical gain medium when doped with rare-earth ions. However, cavities are always needed for lasers. The random distribution of PNCs in glass matrix may offer a potential platform for stable random lasers without cavities (*13*). The tunable bandgap in semiconducting NCs (e.g., PNCs) endows glass with a tunable emission wavelength range that is beyond that of rare-earth ion-doped glass (*14*, *15*). However, continuous-wave (CW) random lasers have never been achieved in the NGCs because of their low grain density for light scattering (*16*).

The stable random lasers are promising devices for various applications, including speckle-free imaging (*17*), spectral measurement with super-resolution (*18*), and networks of coupled resonators (*19*) due to their low coherence, ease of fabrication, and low structural dependence. However, the production of single-mode random lasers with high spectral purity and quality is still a challenge, and complex excitation design is commonly necessary for manipulating the modes of random lasers (*20*). For example, the wave-front shaping of pump beam by spatial light modulators (SLMs) or digital micromirrors based on the excitation design has been widely developed to induce single-mode lasing (*21*, *22*). Typically, the highly complex illumination patterns and precisely designed cavities are required for stable CW single-mode lasers, which hinder various applications including the on-chips integration of laser devices, photonic circuits, and flexible laser for wearable devices.

In this study, we report the modulation of the photoluminescence (PL) and lasing properties of PNCs in glass at temperatures considerably lower than *T*_g_ for a processing short time. We developed low-temperature thermal strategies to precisely control the nanophase separation and ion distribution in PNCs. We proposed a mechanism for the scattering enhancement, and demonstrated a CW single-mode random lasing by constructing PNCs-in-glass hierarchical structure. We achieved the stable flexible laser by incorporating the hierarchical structures into the polydimethylsiloxane (PDMS) film.

## RESULTS

### Regulation and material characterization of the NGCs

There are two separated phases in NGCs: glass phase and NCs phase (*10*). The melting temperature (*T*_m_) of the NCs can be much lower than *T*_g_. Hence, ion exchange, nanophase separation, and crystallization in the perovskite domains can be exploited to modulate the properties of NCs and, consequently, NGCs at temperatures below *T*_g_. This represents an innovative approach to manipulate the optics of NGCs.

**Fig. 1.**
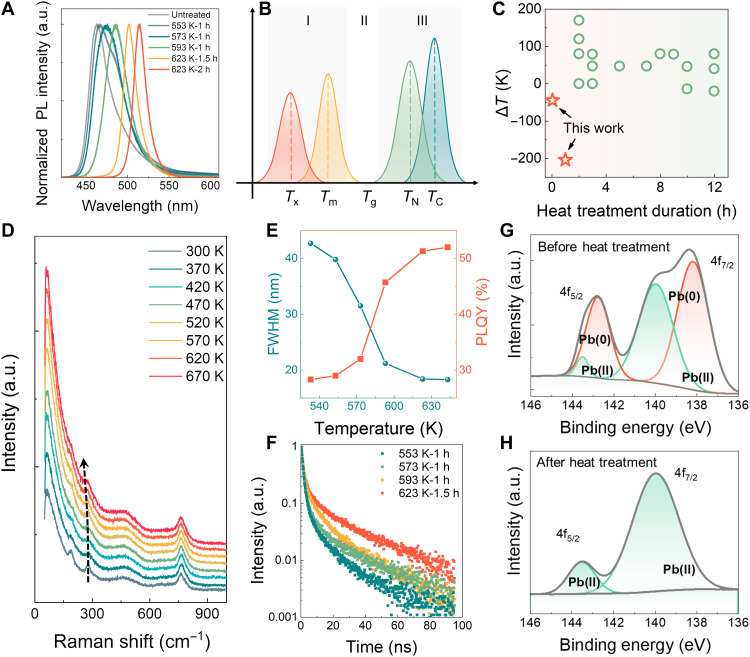
The PL regulation of perovskite NGCs at temperatures below *T*_g_. (**A**) The corresponding PL spectra cover the region from 465 to 515 nm of CsPb(Cl_1-*x*_Br*_x_*)_3_ PNCs under different heat treatment conditions. The samples with component G1 are detailed in table S2. (**B**) Region I represents the heat treatment temperatures used in this work; region II represents the *T*_g_, and region III represents the heat treatment temperatures used in previous works for precipitating crystals in glass, where *T*_x_, *T*_m_, *T*_g_, *T*_N_, and *T*_C_ represent the temperatures of heat treatment, grain melting, glass transition, nucleation, and crystal growth, respectively. (**C**) Summary of the reported Δ*T* (*T*_x_ - *T*_g_) and heat treatment duration in the process of preparation and optimization of perovskite NGCs. (**D**) In situ Raman spectra of NGCs under temperatures from 300 to 670 K. (**E**) The FWHM and PLQY of perovskite NGCs under heat treatment temperatures from 553 to 643 K for 1 hour. (**F**) Time-resolved PL decays of perovskite NGCs under different heat treatment conditions. The lifetimes under the heat treatments at 553, 573, and 593 K for 1 hour and 623 K for 1.5 hours are 6.54, 7.99, 8.15, and 13.25 ns, respectively. (**G** and **H**) XPS spectra of perovskite NGCs (G) before and (H) after thermal activation. h, hours. a.u., arbitrary unit.

To implement this approach, Cl–Br codoped perovskite NGCs with separated phases are prepared. The Cl^−^ ions, compared to Br^−^ ions, have a lighter atomic mass, smaller ionic size, and stronger complexation affinity with Pb^2+^ ions, thereby facilitating the preferential aggregation and precipitation of Cl-rich PNCs, which emit at 465 nm during the cooling process (*8*). Two phases could be created in the NC domain after the quenching process: Cl-rich PNC phase and amorphous Br-rich phase (*23*). The increase of the heat treatment temperature and time promotes the diffusion of the Br^−^ ions and enables the substitution of Cl^−^ ions by Br^−^ ions owing to the chemical potential gradient, thereby tuning the composition of CsPb(Cl_1-*x*_Br*_x_*)_3_ PNCs (*8*, *12*). The PL of CsPb(Cl_1-*x*_Br*_x_*)_3_ NCs can be continuously tuned in the wavelength range from 465 to 515 nm upon heat treatment at low temperatures (*T*_x_) well below *T*_g_ (735 K, the glass transition temperature of the primary glass phase without halide elements; fig. S1), as shown in Fig. 1A. Both x-ray diffraction (XRD; fig. S2A) and transmission electron microscopy (TEM; fig. S2, B to E) confirm the presence of CsPb(Cl_1-*x*_Br*_x_*)_3_ NCs after heat treatment. The lattice fringes in the high-resolution TEM (HRTEM) images (fig. S2, B and D) are assigned to the (200) and (110) crystal planes of the CsPbX_3_ unit cell, respectively. The upper limit of the loading fraction of NCs in the glass matrix is about 18% (wt) based on the chemical composition of the initial glass. The TEM images also confirmed that the NCs in glass have a relatively uniform distribution. This is reasonable since the PNCs were precipitated in the uniform and isotropic glass phase, and the aggregation was prevented by the stable glass matrix. The mean sizes of PNCs heat treated at 533, 553, 573, and 623 K for 1 hour are approximately 18.6, 20.56, 24.8, and 28.1 nm, respectively (fig. S3), which are considerably larger than the Bohr diameter of CsPb(Cl_1-*x*_Br*_x_*)_3_ (*24*). In addition, CsPbBr_3_ PNCs maintain their PL peak positions at 513 nm under different heat treatment conditions, further confirming that composition engineering, rather than the quantum size effect, is responsible for tuning the PL of heat-treated perovskite NGCs (fig. S4). The lowest heat treatment temperature (*T*_x_) of 533 K is 90 K below the *T*_m_ (623 K) of PNCs and 202 K below *T*_g_ (735 K) of the glass phase (Fig. 1B and fig. S1), which is much lower compared to the reported values (Fig. 1C and table S1). All the adopted *T*_x_ values are lower than *T*_g_, and the heat treatment time is shorter than previously reported values. We also confirm the suitability of this strategy for other glass systems to regulate the PL wavelength of PNCs (fig. S5 and table S2). Therefore, our strategy enables the manipulation of the PL at lower temperatures and shorter processing time in perovskite NGCs compared to conventional approaches, thereby saving processing energy.

In situ Raman measurements further confirm the ion exchange process during heat treatment (Fig. 1D). The Raman peak at 286.4 cm^−1^ is attributed to the second-order combination of transverse and longitudinal optical phonons (TO_3_ + LO_3_) of CsPb(Cl_1-*x*_Br*_x_*)_3_ PNCs, verifying the formation of PNCs (*25*, *26*). The Raman band remains unchanged as the temperature increases from 300 to 470 K. An obvious red shift occurs with a further increase in temperature from 520 to 670 K, indicating that Br^−^ ions start replacing Cl^−^ ions in the NCs (*27*). The Raman band at 191 cm^−1^ is associated with the motion of Cs^+^ ions in CsPb(Cl_1-*x*_Br*_x_*)_3_ NCs (*26*) and gradually broadens with increasing the heat treatment temperature and then disappears at 620 K, indicating that the crystal structure of CsPb(Cl_1-*x*_Br*_x_*)_3_ NCs becomes unstable as the temperature increases. Last, the NCs melt and decompose at 620 K. Heat treatment below *T*_m_ of CsPb(Cl_1-*x*_Br*_x_*)_3_ NCs (520 K–620 K) is a key factor for ion exchange, which is consistent with PL tuning.

The PLQY of the PNCs increases, and the full width at half maximum (FWHM) decreases with increasing the temperature (Fig. 1E). The highest PLQY at 515 nm is 52.1%. The decrease in FWHM could result from the improvement of crystal quality confirmed by the increase of QY and the increase of Br^−^ in the CsPb(Cl_1-*x*_Br*_x_*)_3_ PNCs. The lifetimes of these PNCs increase as shown in Fig. 1F, indicating a reduction in nonradiative recombination. The calculated exciton binding energy (*E*_b_, 106 meV) exceeds the previously reported values for both perovskite NGCs and colloid PNCs (fig. S6) (*8*, *24*, *28*), indicating a higher probability of radiation recombination (*29*). The Pb 4f x-ray photoelectron spectroscopy (XPS) spectrum of untreated perovskite NGCs is deconvoluted into four peaks (Fig. 1G). The two peaks at 138.1 and 142.7 eV are attributed to Pb^0^ defects in its metallic state (*30*). Heat treatments result in the disappearance of these defects (Fig. 1H), which is responsible for the increase of PLQY. The slight differences in the peak shape and FWHM of Pb(II), as shown in Fig. 1, G and H may arise from several factors such as the overall change of the valence state of Pb ions before and after heat treatment and slight variations in sample surface homogeneity. This phenomenon commonly occurred in NGCs (*31*). First-principle density functional theory (DFT) calculations imply that the repair of halide vacancy defects during heat treatment could eliminate the trap states between the conduction band and valence band (fig. S7), thereby also increasing the PLQY. It can be concluded that low-temperature heat treatment (below the *T*_m_ of PNCs) facilitates ion diffusion in PNCs and repaired defects, including Pb^0^ and halide defects (*32*, *33*). On the basis of the solid-state ion exchange strategy, we modulate the optical properties of PNCs by heat treatment at lower temperatures (below *T*_g_) for shorter processing time (1 hour) compared to the techniques reported previously (*9*, *10*). The continuous PL tuning and high PLQYs are achieved in NGCs.

### The preparation of PNCs-in-glass hierarchical structure

**Fig. 2.**
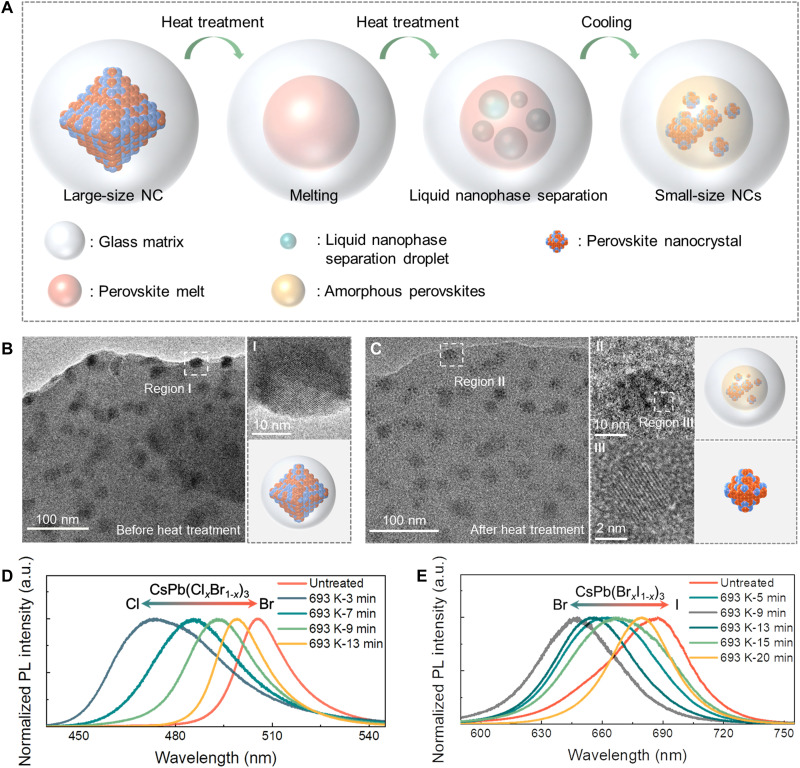
The formation and regulation of the PNCs-in-glass hierarchical structure. (**A**) The schematic diagram of the formation of PNCs-in-glass hierarchical structure. (**B**) TEM image of PNCs before heat treatment. Inset: The HRTEM image exhibits a whole large-size crystal grain in region I. The lattice fringes in region I are assigned to the (220) crystal planes of the CsPbX_3_ unit cell. (**C**) TEM image of PNCs-in-glass hierarchical structure after heat treatment at 693 K for 7 min and subsequent quenching. Inset: Region II exhibits the HRTEM image of small-size dispersed PNCs in a large-size amorphous perovskite nanoparticle. Region III is the HRTEM image of a single PNC in region II. The lattice fringes in region III are assigned to the (110) crystal planes of the CsPbX_3_ unit cell. (**D**) The corresponding PL spectra cover the region from 473 to 505 nm of CsPb(Cl_1-*x*_Br*_x_*)_3_ PNCs under different heat treatment conditions and subsequent quenching. Untreated samples in (D) are obtained by treating samples with component G1 at 693 K for 1.5 hours. (**E**) The corresponding PL spectra cover the region from 645 to 687 nm of CsPb(Br_1-*x*_I*_x_*)_3_ PNCs with component G4 (table S2) under different heat treatment conditions and subsequent quenching.

By taking advantage of the difference in size and chemical potential between Cl and Br ions, the halide composition and structure of heat-treated PNCs could also be continuously regulated by tuning heat-treatment temperature slightly above *T*_m_ of the PNCs but lower than the *T*_g_ of the primary phase, for a short heat treatment time (down to several minutes) (Fig. 2A). Unlike the above-discussed all-solid-state process, the large PNC grains separated from the glass matrix (Fig. 2B) gradually melt during heating, and Cl-rich liquid nanophases can be generated first, facilitated by the rapid Cl^−^ ion migration. By precisely controlling the heat treatment duration, the Br^−^ ions gradually diffuse into the Cl-rich domains and Cl^−^ ions move out in an opposite direction, driven by a chemical potential gradient, thereby regulating the halide composition in the PNCs (*8*). As shown in Fig. 2D, the emission of the PNCs is tuned in the range 473 to 505 nm under different heat treatment conditions. In contrast, several hours are usually required for solid-phase separation and ion exchange in the traditional heat treatment strategy (*9*,  *10*, *14*). Small-sized dispersed PNCs are produced in the large amorphous perovskite nanoparticles, which result from the freezing of the melt perovskite domains driven by rapid quenching, leading to the formation of a previously unknown type of NGCs: PNCs@amorphous perovskite nanoparticle@glass hierarchical structure, named as PNCs-in-glass hierarchical structure (Fig. 2C and fig. S8, A to C). The energy dispersive spectroscopy (EDS) results confirm that the amorphous matrix encapsulating PNCs contains halide ions, Pb^2+^ and Cs^+^ (fig. S9). The mean size of the dispersed PNCs under different heat treatment conditions remains almost unchanged (between 4.10 and 4.25 nm; fig. S8, D to F), indicating that the composition engineering, rather than quantum size effect, is primarily responsible for tuning the PL of PNCs. In addition, no sub-bandgap emission, which may be associated with the amorphous perovskite, is observed in PL spectra, confirming that the PL emission originates from the PNCs rather than from the amorphous perovskite nanoparticles (*34*). The sizes of the amorphous perovskite nanoparticles under various heat treatment conditions are comparable to those before heat treatment, suggesting minimal interaction between the PNCs and the glass matrix due to both the processing temperatures being lower than the *T*_g_ and a short processing duration (several minutes) (fig. S8, G to I).

The proposed principle is universal to tune the PL of CsPb(Br_1-*x*_I*_x_*)_3_ NCs and PNCs in other glass systems. For example, the PL spectra of CsPb(Br_1-*x*_I*_x_*)_3_ in PNCs-in-glass hierarchical structures are successfully tuned from 647 to 688 nm in 20 min (Fig. 2E). Regulating the PL wavelength of PNCs in the borate and borophosphate glasses is also achieved (fig. S10 and table S2).

### Random lasing based on the scattering-enhancement strategy

**Fig. 3.**
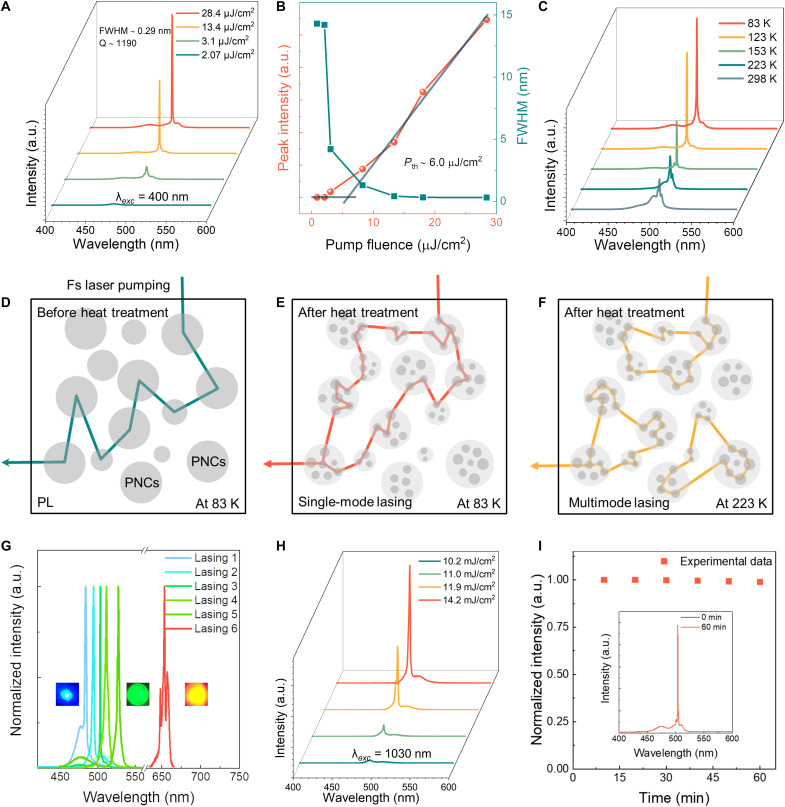
High-quality single-mode lasing pumped by a femtosecond laser. (**A**) Power-dependent emission spectra from the PNCs-in-glass hierarchical structure prepared by heat treatment at the temperature of 693 K for 3 min. Narrow emission peaks at ∼503 nm are indicative of lasing. (**B**) Peak intensity and FWHM of the emission spectra as a function of pumping fluence (*P_th_* ~ 6.0 μJ/cm^2^). (**C**) Temperature-dependent emission spectra. (**D** to **F**) The schematic diagram of multiple scattering in perovskite NGC and PNCs-in-glass hierarchical structure under different temperatures. (**G**) The emission spectra in the range from 484 to 527 nm and 656 nm. Lasing 1: FWHM ~1.2 nm@484-nm lasing; lasing 2: FWHM ~0.61 nm@495-nm lasing; lasing 3: FWHM ~0.29 nm@503-nm lasing; lasing 4: FWHM ~0.59 nm@511-nm lasing; lasing 5: FWHM ~0.47 nm@527-nm lasing; lasing 6: FWHM ~0.8 nm@656-nm lasing. All the spectra were recorded at 83 K. The insets are the optical images of lasers at 484, 527, and 656 nm in PNCs-in-glass hierarchical structures. (**H**) Power-dependent emission spectra from the PNCs-in-glass hierarchical structure pumped by a 1030-nm femtosecond laser. (**I**) Peak intensity for perovskite PNCs-in-glass hierarchical structure under constant pulsed excitation for 60 min. The inset shows the emission spectra before and after 60 min of constant pulsed excitation.

The PNCs-in-glass hierarchical structure with small PNCs encapsulated in a large amorphous nanoperovskite matrix provides an ideal platform for random lasers. In this case, the increase in the density of the NCs provides enhanced light scattering and optical gain for high-quality lasers. We pumped the as-prepared hierarchical structure using a 400-nm femtosecond laser. Spontaneous emission centered at 475 nm with a broad FWHM (~14 nm) is observed under low-fluence pumping. A sharp peak appears at 503 nm when the pumping fluence exceeds the lasing threshold, indicating the generation of single-mode lasing, which is further supported by the enlarged lasing peak spectrum (Fig. 3A and fig. S11). To accurately assess the intrinsic optical quality of the cavity, the quality factor (Q) was calculated under near-threshold conditions with the FWHM (∆λ) of the lasing spectrum of as small as 0.29 nm, which was controlled by photon lifetime and cavity loss rather than gain-related noise. The Q factor reaches 1190 calculated with an equation of λ/∆λ, where λ is the lasing wavelength. To the best of our knowledge, this is an innovative study demonstrating single-mode lasing in NGCs. The pumping threshold is determined to be approximately 6.0 μJ/cm^2^ (Fig. 3B). Figure 3B also reveals that the emission intensity increases relatively slowly at low pump fluence and faster at high pump fluence, which may be due to the stronger influence of scattering loss and decrease of possibility of coherent random feedback in the former case and have been observed in the random lasing or amplified spontaneous emission (*35*–*37*). Higher pump fluence leads to stronger coherent random feedback with fast increase of lasing intensity (*38*). Single-mode lasing changes into multimode lasing at 223 K (Fig. 3C). Random lasing occurs even at room temperature.

Creating strong scattering and obtaining sufficient optical gain are essential for realizing random lasers (*13*, *39*). In the case of femtosecond laser pumping, the optical gain threshold, given by $I_{\text{flu}}=1.15\;{\mathrm{h\nu}}_{p}/\sigma_{\text{abs}}$, where $I_{\text{flu}}$ is the pulse fluence, ${\mathrm{h\nu}}_{p}$ is the pump photon energy and $\sigma_{\text{abs}}$ is the absorption cross-section of PNCs, is not directly affected by the nonradiative recombination, particularly the Auger recombination, which is primarily responsible for the decay of multiexcitons (see discussions in text S1) (*40*). No lasing occurs in the initial non-heat–treated perovskite NGCs, indicating the critical role of the unique PNCs-in-glass hierarchical structure for light scattering. On the basis of our findings, we propose a scattering enhancement mechanism for the creation of random lasers in the PNCs-in-glass hierarchical structure detailed in text S2. The scattering strength (*I*_s_) in the glass matrix can be described (*41*): $I_{s}\propto \frac{2\pi \rho }{k^{4}}\int_{0}^{2k}F\left(q\right)S\left(q\right)\mathrm{qdq}$, where ρ is the particle density, *k* is the wave vector in the glass, the *F*(*q*) is the form factor (single-particle scattering function), and the *S*(*q*) is the structure factor (collective interaction). The particle density ρ increases significantly after the heat treatment (Fig. 3, D and E), which helps to improve the construction of spatial correlation based on the structure factor *S*(*q*) and induce the formation of the high-quality modes (*20*). The *I*_s_ is significantly improved, enabling random lasers with incoherent feedback along open paths and coherent feedback along closed loops initially in the PNCs-in-glass hierarchical structure (*38*). Consequently, the dominance of closed loops is realized with continuously increasing pump laser energy, exhibiting the distinct localization of photons (Fig. 3E) (*42*). However, two closed loops with significant spatial overlap cannot lase simultaneously because of the gain competition, and, in other words, distinct lasing mode must be spatially separated (*43*). Hence, as shown in Fig. 3E, a single-mode random lasing is generated in a dominant low-loss closed loop with a low *P*_th_ when the excitation spot diameter is appropriately controlled (~50 μm in the current case). Further increasing the excitation spot diameter (e.g., diameter ~100 μm) enhances the scattering area, leading to the formation of multiple spatially separated closed loops (fig. S12A). Consequently, as illustrated in fig. S12B, the lasing behavior transforms from single-mode to multimode, agreeing with the previous report (*44*). In another case, when the spot diameter is too small, for example ~20 μm, no lasing occurs. The weak (shoulder) peak in Fig. 3A may be attributed to a lasing mode with small light amplification. Unfortunately, it is too weak to be discussed in detail at present. More work is needed to uncover the origin.

With an increase in temperature, the enhanced exciton-phonon interaction leads to a considerable optical loss during the scattering process, signifying a shorter transport light path, thereby resulting in formation of more closed loops rather than single closed loop within the same excitation area (Fig. 3F) (*45*). Hence, a higher random lasing threshold is required to achieve sufficient gain to compensate for the optical loss at 223 K (fig. S13) (*39*). The increase in the mode number induced by more closed loops ultimately leads to the transition from single-mode to multimode lasing (Fig. 3C) (*43*). On the basis of this scattering-enhancement strategy, single-mode random lasers with tunable wavelengths in the range 484 to 527 nm and a multimode lasing with a wavelength of 656 nm are achieved (Fig. 3G and figs. S14 to S16), which is beyond the achievement in the traditional rare-earth ion-doped glass. The relatively unstable crystal structure and lower *E*_b_ of CsPb(Br_1-*x*_I*_x_*)_3_ PNCs compared to those of CsPb(Cl_1-*x*_Br*_x_*)_3_ NCs results in the enhanced exciton-phonon scattering and exciton dissociation, leading to a considerable optical loss even at *T*_x_, similar to the random lasing at high temperature (*46*, *47*). Thus, it is challenging to establish a single closed-loop coherent path. A multimode lasing is obtained (Fig. 3G). Single-mode lasing is also realized when the sample is excited using a 1030-nm femtosecond laser via three-photon pumping with a threshold of approximately 10.8 mJ/cm^2^ (Fig. 3H and fig. S17). It also changes to multimode lasing at room temperature, which can be explained according to the mechanism discussed above (fig. S18). The present PNCs-in-glass hierarchical structures are not simple mechanical mixing composites as shown in the most reported results. They allow for unique light-scattering behavior and single-mode lasing action. In addition, no cavities or mode-selection components are necessary in the current single-mode lasers, and this is pivotal for device integration with a minimum size.

Owing to the protection of the glass, lasing in PNCs-in-glass hierarchical structure exhibits excellent stability with no obvious change in the lasing spectrum after irradiation for 60 min (corresponding to ~4.5 × 10^7^ pulses) as shown in Fig. 3I. The hierarchical structure is also stable after immersion in ethanol for 9 months, 20 heating-cooling cycles, and 240 min of continuous ultraviolet laser irradiation (fig. S19). This high stability enables their long-term practical applications.

### Demonstration and characterization of CW lasing

**Fig. 4.**
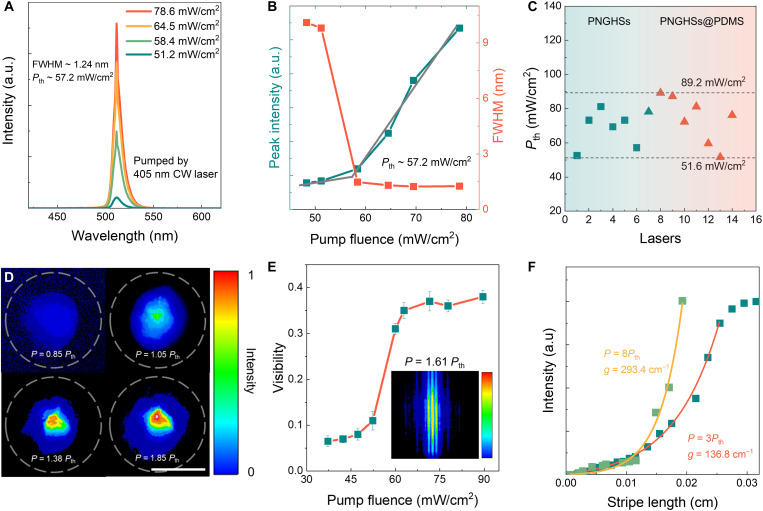
CW single-mode random lasers. (**A**) Power-dependent emission spectra from the PNCs-in-glass hierarchical structure prepared by heat treatment at the temperature of 693 K for 13 min. Narrow emission peaks at ∼511 nm are indicative of lasing at 83 K. (**B**) Peak intensity and FWHM of the emission spectra as a function of pumping fluence (*P*_th_ ~ 57.2 mW/cm^2^) at 83 K. (**C**) Experimental thresholds of lasers from PNCs-in-glass hierarchical structures and PNCs-in-glass@PDMS hierarchical structures. (**D**) The real-space images of the emission spot under different pump fluences ranging from 0.85 *P*_th_ to 1.85 *P*_th_. Scale bar (D), 50 μm. (**E**) The spatial coherence of random lasers under different pump fluences calculated by $V=\left(I_{\text{max}}-I_{\text{min}}\right)/\left(I_{\text{max}}+I_{\text{min}}\right)$, where $I_{\text{max}}$ and $I_{\text{min}}$ are the local maximum and minimum values of the interference intensity near the observation point, respectively. The inset is the interference fringes under the pump fluence of 1.61 *P*_th_. (**F**) The lasing intensities increase as a function of the excitation stripe length under fixed pump fluences of 3 *P*_th_ and 8 *P*_th_, as determined by the variable stripe length method. A supralinear increase confirms the occurrence of the lasing.

The PNCs-in-glass hierarchical structure is further pumped by using a commercial 405-nm CW laser as the excitation source. Figure 4A shows the evolutions of the emission spectra and the peak intensity as a function of the pumping power. A smaller FWHM of the PL spectra was observed compared to the pulsed laser pumping, which is frequently reported for the perovskites and may originate from the stronger optical loss in the CW laser pumping as discussed later (*48*). The lasing peak at 511 nm has an FWHM ~1.24 nm, and the emission intensity increases obviously after the pumping fluence is over the critical value (Fig. 4B). The *P*_th_ reaches 52.6 mW/cm^2^ (Fig. 4C), which is three orders of magnitude lower than the reported values for other CW lasers with cavities (fig. S20). The corresponding carrier density is estimated to be 5.2 × 10^18^ cm^−3^, which is higher than the reported Morr density of the perovskites (*49*). This is reasonable for the following reason. Because of the absence of well-designed cavities, random lasers exhibit considerable cavity loss, which leads to a longer estimated average carrier lifetime, and thus a higher carrier density (typically >10^18^ cm^−3^) is necessary for lasing (text S3) (*50*, *51*). The observed variations in thresholds (ranging from 52.6 to 81.2 mW/cm^2^ in Fig. 4C) are attributed to slight fluctuation in the distribution of PNCs within glass matrix. The presence of a distinct threshold in terms of both output intensity and linewidth (Fig. 4B), the beam profiles (Fig. 4D), and the spatial coherence (Fig. 4E and fig. S21) clearly confirm the achievement of lasing (*52*–*54*). The optical gain of PNCs-in-glass hierarchical structure increases from 136.8 cm^−1^ under 3*P*_th_ to 293.4 cm^−1^ under 8*P*_th_ (Fig. 4F), which is higher than that of the previous reported CW lasers (*55*) and is consistent with the low lasing threshold. The progressively slower rate of the intensity increase in Fig. 4F is attributed to the gain saturation effect.

The characteristics of no cavities, CW laser excitability, and low *P*_th_ of lasers in hierarchical structure endow them with high potential as the flexible laser devices. To verify this proposition, the PNCs-in-glass hierarchical structures are incorporated in PDMS film for the demonstration of the flexible lasing (fig. S22A). The prepared PNCs-in-glass@PDMS hierarchical structures exhibited remarkable flexibility as shown in fig. S22B. The *P*_th_ of flexible laser is determined to be 51.6 mW/cm^2^. The lasing peak appears at 511 nm with an FWHM ~1.54 nm under a 405-nm CW laser excitation (fig. S22, C and D). The samples still retain the ability to generate CW laser at 123 K, a temperature approximately 40 K above the *T*_g_ of PDMS, thereby preserving their intrinsic flexibility (fig. S22E). Notably, the lasing properties at different positions from the same PNCs-in-glass@PDMS film remain consistent (fig. S23), confirming the high repeatability and stability of the flexible lasing properties. The lasing in the PNCs-in-glass@PDMS hierarchical structure also exhibits the promising stability under a consistent excitation by a 405-nm CW laser, and no obvious change is observed in the lasing spectrum after irradiation for 60 min as shown in fig. S24. The wavelengths of CW lasers are tunable through regulating the composition of the PNCs-in-glass hierarchical structures as shown in fig. S25. The CW lasers, which are originated from both PNCs-in-glass hierarchical structure (fig. S26) and that dispersed in PDMS (fig. S22E), directly evolves to the spontaneous emission when further increasing the temperature above 123 K, and no multimode lasing is observed. This is because the strong thermal effect induced by CW laser pumping causes damage to the PNCs before reaching the *P*_th_ of the multimode CW lasing.

We observe that the narrow lasing peak emerges at the maximum of the PL spectrum when the pump fluence exceeds *P*_th_, being consistent with the characteristics of the incoherent optical feedback (*38*, *56*). In the case of CW laser pumping, the optical gain threshold is given by $I_{\text{int}}=h\nu_{p}/\left[\sigma_{a}{\left(\tau_{A,\mathrm{XX}}\tau_{X}\right)}^{1/2}\right]$, where $\tau_{A,\mathrm{XX}}$ is the bixeciton Auger lifetime and $\tau_{X}$ is the single-exciton lifetime (see text S3) (*50*). Different from the case of femtosecond laser pumping, the gain threshold under CW laser pumping involves the steady-state process of multiexciton generation and decay, thereby making the effects of both Auger recombination and thermal phenomena notable and nonnegligible (*50*, *57*). The incoherent feedback feature of the current CW random lasers could be attributed to the effect of optical loss (text S3). Under CW pumping, it is challenging to establish a single, independent mode for coherent feedback, unlike in the case of femtosecond laser pumping. Instead, coupled modes with strong interactions are formed, allowing photons to exchange among these modes (fig. S27). Therefore, as the pump fluence increases, single-mode lasing emerges at the maximum of the PL spectrum only when the optical gain at the frequency near the maximum of the gain spectrum is sufficient to compensate for the losses across all coupled modes. With further increase in pump fluence, the strong interaction among coupled modes enables the photo exchange toward this amplified mode and prevents photon accumulation in other modes. In addition, the thermal effect induced by the CW pump laser may also lead to increased optical loss, ultimately resulting in the emergence of a single-mode incoherent lasing against a higher PL background. This incoherent feedback leads to a relatively larger FWHM (1.24 nm) in the lasing spectrum compared to the coherent random lasing with the FWHM of 0.29 nm (*38*, *56*).

### Applications using stable CW lasing

**Fig. 5.**
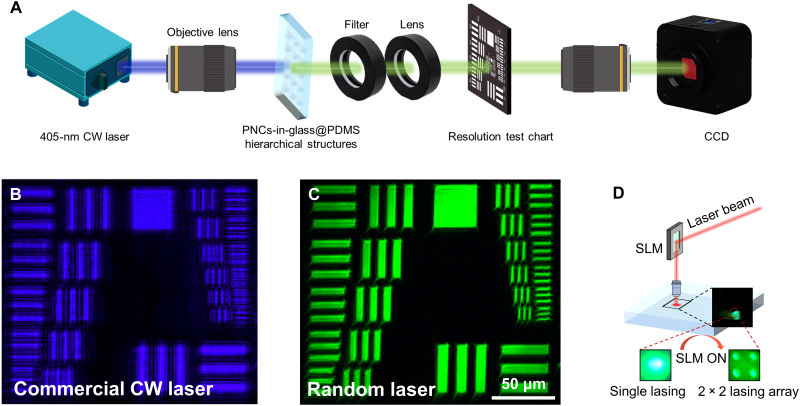
Random lasers for speckle-free images and lasing array. (**A**) Schematic illustration of experimental setup for speckle-free laser imaging. (**B** and **C**) Speckle-free images illuminated by a 405-nm commercial CW laser and random laser using a 1951 US Air Force resolution test chart, respectively. (**D**) Demonstration of 2 × 2 lasing array achieved by an SLM. The pixel pitch among the lasing array is only 7.5 μm.

The random lasers, characterized by their low spatial coherence, have been demonstrated to be a promising illumination source for optical imaging (*17*), which can achieve superior image quality compared to the commercial CW laser imaging based on experimental setup for speckle-free laser imaging as shown in Fig. 5A, further validating the occurrence of random laser with incoherent feedback nature (Fig. 5, B and C). The stable, repeatable, low *P*_th_ and no cavities of lasing emissions demonstrate the high potential of the PNCs-in-glass@PDMS hierarchical structure as flexible lasing for next-generation wearable devices. The stable lasing output demonstrates the potential of the hierarchical structure as for lasing devices. As a proof of concept, holography based on a liquid-crystal SLM is applied to display a 2 × 2 lasing array by loading a computer-generated hologram (Fig. 5D). A pixel pitch of only 7.5 μm within the lasing array enables the high-resolution laser display. The dynamic holographic display of “a running dog” is demonstrated in fig. S28 and movie S1 via an SLM.

The above-presented mechanisms of the formation of PNCs-in-glass hierarchical structure could be universal to various types of NCs and quantum dots (e.g., CdS*_x_*Se_1-*x*_) and create other hierarchical structures in glass for diverse applications (*10*). Furthermore, such mechanism is also applicable to other multiple-phase systems such as ceramics, resins, gels, polymers, and metal-organic framework (*58*–*62*). New hierarchical structures, which may exhibit unique properties different from the initial systems, could enable new functionalities and applications. For the applications, we demonstrate that random lasers can be used for display based on the dynamic holographic display technique, thereby expanding the applications of random lasers. This dynamic holographic display benefit from the fabrication flexibility of random lasers. Moreover, the low spatial coherence of our random lasers not only allows for speckle-free laser imaging but also for bright eye-friendly laser display (*63*).

## DISCUSSION

In summary, we have revealed a previously undiscovered general technique to tune the optics of perovskite NGCs via energy-efficient thermal strategies. The internal mechanisms are revealed. In particular, the PNCs-in-glass hierarchical structure is produced and shows stable single-mode random lasing using both femtosecond laser and CW laser as the excitation sources with ultralow thresholds. The lasing wavelengths are tunable. A scattering-enhancement mechanism is proposed for generating random lasing. The speckle-free laser imaging and dynamic holographic display are achieved. The stable and repeatable flexible laser with a low threshold is demonstrated, showing strong potential for wearable device application.

## MATERIALS AND METHODS

### Sample preparation

SiO_2_ (99.99%), H_3_BO_3_ (99.99%), CaO (99.99%), ZnO (99.99%), Na_2_CO_3_ (99.99%), PbCl_2_ (99.99%), NaCl (99.99%), NaBr (99.99%), and Cs_2_CO_3_ (99.99%), were purchased from Aladdin (Shanghai, China). All chemicals were used without any further purification. Synthesis of CsPb(Cl_1-*x*_Br*_x_*)_3_ PNCs: A series of mixed-halide perovskite PNCs were prepared via conventional melting-quenching method and subsequent heat treatment. The original raw material mixtures were designed with the molar ratio in table S1, ground into powders in an agate mortar, and then kept in closed corundum crucibles and sintered in a muffle under an ambient atmosphere at 1473 K for 15 min. The melt was poured onto a preheated stainless steel plate for quenching and pressed with a brass plate to form precursor glass samples with a thickness of 2 mm. All samples were cut and polished to the optical grade, and some of them were ground as fine powders for the next experimental characterizations and ultrafast laser patterning.

### Characterization

XRD patterns were acquired by using x-ray diffractometer (Cu Kα, λ = 1.5406 Å; PANalytical B.V., Empyrean 200895). The TEM and HRTEM images were taken on a transmission electron microscope (JEOL, JEM-2100F) at 200-kV accelerating voltage. XPS spectra were obtained by Escalab 250Xi spectrometer. The in situ Raman spectra of PNCs were obtained by micro confocal Raman spectrometer (RENISHAW, Invia) equipped with a 405-, 532-, and 785-nm wavelength CW laser with a temperature-controlled plate for continuous heating. The PLQY of the PL was measured using a PLQY spectrometer (Hamamatsu, Quantaurus-QY Plus C13534-12). The PL decay curves of PNCs were carried out with a luminescence spectrometer (Edinburgh Instruments FLSP920, England). The heat flow curve for the original glass sample was measured during upscan at 10 K/min in argon by using a differential scanning calorimeter.

### Lasing measurements

Optically pumped lasing measurements were carried out with a homebuilt confocal fluorescence spectrophotometer setup with optical multichannel gratings (150/600/1800 g/mm, the test step length is about 0.03 nm). The temperature of samples for pumped lasing measurements was decreased to −190°C with the support of liquid nitrogen. The 1030- and 400-nm pulsed lasers (95 fs, 22 kHz) as excitation light were focused onto the NGCs surface by using 10×, 20×, and 50× objective (numerical aperture = 0.3, 0.45, and 0.75). The PL spectra under different temperatures were obtained from a homebuilt confocal fluorescence spectrophotometer excited by 405-nm commercial CW laser (Q-lite-405-20-SM, Raytum Photonics, USA) with a temperature-controlled plate for continuous heating or cooling. The tuning of *T*_x_ was achieved by filling liquid nitrogen into the temperature-controlled plate. Holographic dynamic display and femtosecond laser arrays were achieved through an SLM (Holoeye PLUTO-2.1 UV-099).

### Computational details

The structural and electronic properties of the CsPb(Cl_1-*x*_Br*_x_*)_3_ PNCs were calculated by using the first-principles DFT and the projector augmented wave method implemented in the Vienna Ab initio simulation package. The generalized gradient approximation of the exchange-correlation potential in the form of the Perdew-Burke-Ernzerhof was used for the calculations throughout this work. The structural models for the CsPb(Cl_1-*x*_Br*_x_*)_3_ PNCs were built as a cubic 3a by 3b by 3c supercell for the subsequent calculations as shown in fig. S7. The plane wave cutoff energy for the expansion of wave functions was set at 400 eV. The conjugate-gradient algorithm was used in all structure models to fully relax the internal forces until energy differences were less than 0.01 meV, and stress tensors were less than 0.02 eV/Å.

## Supplementary Materials

### The PDF file includes:

Supplementary Text Figs. S1 to S28 Tables S1 and S2 Legend for movie S1 References
