# Supplementary material for: Perovskite nanocrystals-in-glass hierarchical structures enable stable continuous-wave random lasers
Source: Sci Adv. 2026 Jan 16;12(3):eadz8460. doi: 10.1126/sciadv.adz8460 (PMC12810581; doi:10.1126/sciadv.adz8460)
Supplement: Supplementary file 1 — Supplementary Text Figs. S1 to S28 Tables S1 and S2 Legend for movie S1 References [file sciadv.adz8460_sm.pdf]

Science Advances
AAAS

Supplementary Materials for

Perovskite nanocrystals-in-glass hierarchical structures enable stable continuous-wave random lasers

Xinkuo Li *et al.*

Corresponding author: Dezhi Tan, wctdz@zju.edu.cn

Sci. Adv. **12**, eadz8460 (2026)
DOI: 10.1126/sciadv.adz8460

The PDF file includes:

Supplementary Text
Figs. S1 to S28
Tables S1 and S2
Legend for movie S1
References

Other Supplementary Material for this manuscript includes the following:

Movie S1

Supplementary Text S1: Analysis of optical gain mechanism of the generation of random lasers pumped by fs laser.

In in perovskite PNCs, the three-level system has been confirmed as a proper model to quantify the effect of the optical gain of the PNCs (*40*). their actual occupation probabilities are connected by the equation:

$$P_0+P_X+P_{XX}= 1 \quad (1)$$

$$P_0=p_0;\ P_X=p_1;\ P_{XX}=\sum_{i=2}^{\infty} p_i =1-p_0-p_1 \quad (2)$$

Where, P_0, P_X and P_{XX} are the model occupation probabilities of the nondegenerate ground state, 4-fold-degenerate single-exciton state, nondegenerate biexciton state, and p_i is the actual occupation probabilities. When the optical gain threshold is reached, the stimulated emission rate should be equal to the absorption rate.

$$r_e=2\gamma P_{XX}+\frac{\gamma}{2}P_X \quad (3)$$

$$r_a=2\gamma P_0+\frac{\gamma}{2}P_X \quad (4)$$

Where the r_e and r_a are the rates of stimulated emission and absorption, and γ the rate of a single spin-allowed CB–VB transition. It can be deduced that the condition of gain threshold is $P_0 = P_{XX}$. In the case of the high photo energy and short optical pulse fs laser pumping, the Poisson statistics can describe the exciton distribution in a PNC (*64*), yielding the I_{flu}=1.15 $h\nu_p/\sigma_a$, where I_{flu} is the per-pulse pump fluence, $h\nu_p$ is the pump photo energy and σ_a is the absorption cross-section. Moreover, Auger decay is much lower than the pumping duration, meaning that the Auger decay dose not influence the optical gain threshold (*50*). However, the enhanced exciton-phonon interaction and thermally activated exciton dissociation caused the significant optical loss with increasing the temperature, meaning that a higher pumping threshold is necessary to compensate the optical loss and leading to a shorten transport light path.

Moreover, higher fs laser pump energy will induce more defects in PNCs, which further increase optical loss caused by the non-irradiative recombination.

Supplementary Text S2: Analysis of light scattering mechanism of the generation of random lasers.

The necessary conditions for the formation of random lasers include: 1. The capacity to generate adequate optical gain; 2. Sufficient light scattering strength. For the first point, PNCs have been confirmed as a promising random laser material to obtain sufficient optical gain due to their high absorption/emission efficiency, large binding energy, bright exciton states, and ultrafast stimulated emission (*2*). In this work, we proposed a new scattering enhancement mechanism through the construction of PNCs-in-glass hierarchical structure for high-quality single-mode random lasers. The PNCs-in-glass hierarchical structure was modeled to analyze the mechanism of random lasing generation, assuming dispersion of hard spheres within the matrix as described for PNCs embedded in glass. The scattering strength (I_s) in the glass matrix can be described (*41*):

$$I_s \propto \frac{2\pi\rho}{k^4}\int_0^{2k} F(q)S(q)qdq \quad (5)$$

Where, q is the scattered wavevector, ρ is the particle density, k is the wavevector in the glass, the $F(q)$ is the form factor (single-particle scattering function) and the $S(q)$ is the structure factor (collective interaction). The number of PNCs in PNCs-in-glass hierarchical structure is much larger than that in non-heat-treated perovskite NGCs, leading to an increase of particle density ρ. Furthermore, higher particle density ρ helps to improve the construction of spatial correlation based on the structure factor $S(q)$ and induce the formation of the high quality modes (*20*). Finally, the scattering strength is significantly improved so that random lasing occurs with resonant feedback along closed paths by recurrent scattering in the PNCs-in-glass hierarchical structure. The transport mean free path (l_t) in heat-treated glass is significantly lower than that in non-heat treated glass, which is determined by (*38*):

$$l_t = \frac{1}{\rho\sigma_t} \quad (6)$$

Where σ_t is the scattering cross-section. The strong scattering of emitted light due to the small mean free-range l_t results in the dominance of closed loops cavity, which exhibits distinct characteristics indicating the localization of photons (*42*). However, attributed to the gain competition, two random cavities with significant spatial overlap could not lase simultaneously, in other words, different lasing modes must be spatially separated (*43*). Hence, a single-mode RL is generated in a low-loss closed loop with a low pumping threshold (P_{th}), which is determined by (*43*):

$$P_{th} \propto \frac{\sqrt{l_t}}{\sqrt{l_a}}\frac{1}{d^{3/2}} \quad (7)$$

Where l_a is absorption length and d is the pump beam diameter. The l_a and d are determined by the glass system and pump light source itself, respectively. In this case, a small l_t will lead to a significant decrease of the P_{th}, which is consistent to our experimental results.

More importantly, the size of PNCs in the hierarchical structure is so small that the strong quantum confinement in PNCs results in a wide separation between electronic states, and then inhibits thermal depopulation of the band-edge electron and hole levels, thereby, further reducing the lasing threshold compared with bulk materials (*16*).

As the diameter of the PNC is much smaller than the wavelength of the light, therefore the Rayleigh scattering dominates the scattering process. The total scattering cross-section of Rayleigh scattering can be express as (*65*):

$$\sigma_t = \frac{8\pi}{3}\left(\frac{2\pi n}{\lambda}\right)^4 r^6\left(\frac{\varepsilon-\varepsilon_0}{\varepsilon+2\varepsilon_0}\right)^2 \quad (8)$$

Where λ is wavelength of the excitation light, n and ε_0 is the refractive index and the relative permittivity of the glass matrix, r and ε is the radius and the relative permittivity of the PNC. By combining formula (6), the transport mean free path is calculated to be 9.7 μm. However, multi-interfaces exist in the PNCs-in-glass hierarchical structure, which could lead to complex and enhanced light scattering with a significant increase in the scattering cross-section. Furthermore, the strong self-absorption of PNCs could also reduce the transport mean free path. Consequently, the simultaneous occurrence of enhanced light scattering and strong self-absorption by PNCs-in-glass hierarchical structure poses a challenge to the accurate experimental measurement of the scattering cross-section and to determine the transport mean free path. When taking actual factors into account, the transport mean free path is expected to be smaller than the above calculated value and more effort is needed to reveal the transport mean free path.

Supplementary Text S3: Analysis of optical gain mechanism of the generation of random lasers pumped by CW laser.

Different from the transient process of fs laser, in the case of the CW pumping, the realization of optical gain requires a steady-state establishment process. We assume that the decay of the double exciton state is compensated by the transition of the single exciton state under the pump of a stable pump rate g, and the decay of the single exciton state is compensated by the transition of the ground state.

$$\frac{P_{XX}}{\tau_{XX}} = gP_{X} \tag{9}$$

$$\frac{P_{X}}{\tau_{X}} = gP_{0} \tag{10}$$

Combined with the condition of gain threshold $P_0 = P_{XX}$, the pump rate at the gain threshold can be derived:

$$g_{gain} = (\tau_{XX}\tau_{X})^{-\frac{1}{2}} \tag{11}$$

Where τ_{XX} is the overall biexciton lifetime and τ_X is the single-exciton lifetime. We assume that the radiative recombination contributes to the single-exciton decay primarily and obtain $\tau_{r,X} = \frac{\gamma}{2} P_X$ and $\tau_{r,XX} = 2\gamma P_{XX}$, which yields $4\tau_{r,XX} = \tau_{r,X}$ based on the statistical consideration, where the $\tau_{r,XX}$ is the radiative biexciton lifetime and the $\tau_{r,X}$is the radiative single-exciton lifetime. The biexciton decay can be expressed by:

$$\tau_{XX} = \frac{\tau_{r,XX} \cdot \tau_{A,XX}}{\tau_{r,XX} + \tau_{A,XX}} \tag{12}$$

Where $\tau_{A,XX}$ is the bixeciton Auger lifetime. The single-exciton lifetime of PNCs is about 15 ns in this work and $\tau_{r,XX} \approx 3.75$ ns , whereas the $\tau_{A,XX}$ is on the order of tens ps, which means that $\tau_{r,XX}$ is two to three orders of magnitude higher than $\tau_{A,XX}$. Therefore, the overall biexciton lifetime is dominated by the Auger recombination, that is $\tau_{XX} \approx \tau_{A,XX}$ according to the equation 11. The optical gain threshold pumped by CW laser can be presented in terms of a pump intensity ($I_{gain} = \frac{h\nu_p g_{gain}}{\sigma_a}$):

$$I_{gain} = \frac{h\nu_p}{\sigma_a \cdot (\tau_{A,XX}\tau_X)^{\frac{1}{2}}} \tag{13}$$

Where $h\nu_p$ is the photon energy of the pump laser, σ_a is the absorption cross-section. The expression indicates that the Auger decay directly influence the optical gain threshold. Moreover, the optical losses during the light scattering and absorption lead to a poor laser performance than that of pumping by fs laser (a wider FWHM and stronger PL background).

We further calculated the effective carrier density $\langle n \rangle$ in the case of CW excitation, which can be expressed as (*66*):

$$\langle n \rangle = \langle N \rangle V_0^{-1} \tag{14}$$

Where the $\langle N \rangle$ is the average number of carrier per PNC and the V_0 is the average volume PNC. $\langle N \rangle = g_{gain}\langle \tau_x \rangle = P_{th}\sigma_a\langle \tau_x \rangle$, where the g_{gain} is pump rate at gain threshold, and $\langle \tau_x \rangle$ is the average exciton lifetime, which can be estimated by $\langle \tau_x \rangle = Q\tau_{r,X}$, where Q is the PLQY of the PNC (*40*). The carrier density under CW excitation at the pump threshold in this work is determined to be about 5.2×10^{18} cm^{-3}. This is reasonable that due to the absence of well-designed cavities, random lasers exhibit significant cavity loss, which leads to a longer estimated average carrier lifetime, and a higher carrier density (typically $>10^{18}$ cm^{-3}) is necessary for CW lasing (*51*).

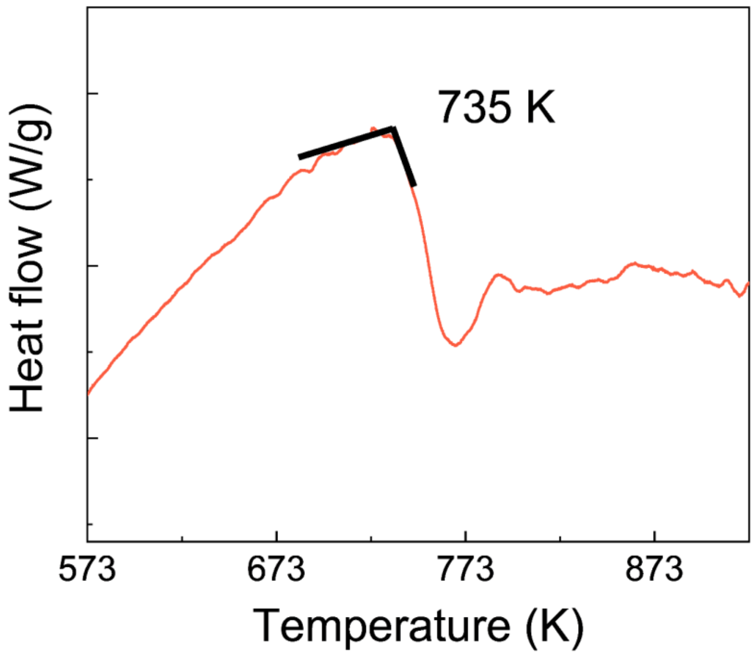

Fig. S1. DSC curve of the glass sample. The glass transition temperature of glass was determined to be 735 K for the glass without doping of halide elements.

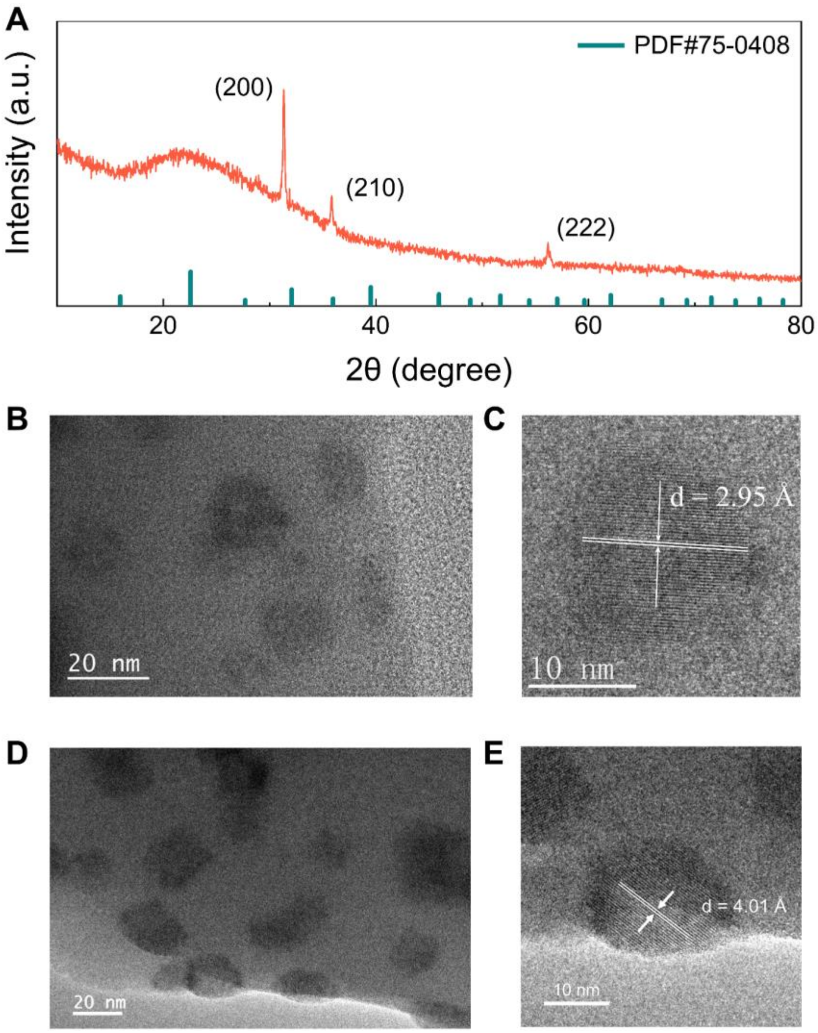

Fig. S2. TEM, HRTEM and XRD images. (**A**) X-ray diffraction pattern of perovskite NGCs prepared at 693 K for 1.5 h. The peaks in the X-ray diffraction pattern can be indexed to the perovskite phase (PDF#75-0408) and the slight shift of the peaks is attributed to the lattice distortion, as they originate from the halide mixed perovskites $CsPb(Cl_{1-x}Br_x)_3$. TEM and HRTEM images of perovskite NGCs heat-treated at (**B**)-(**C**) 553 K and (**D**)-(**E**) 623 K for 1 h. The observed crystalline lattice fringes can also be assigned to the (200) and (110) planes of perovskites, respectively.

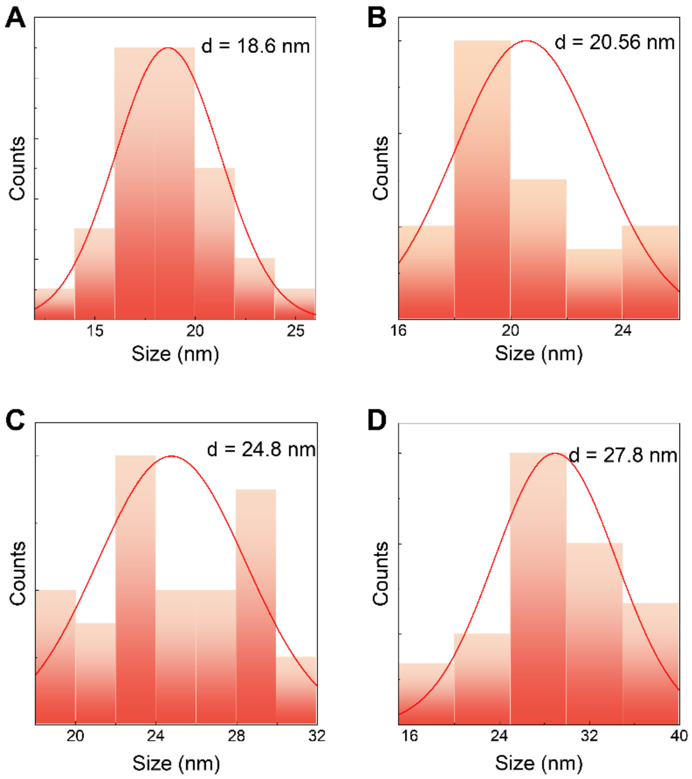

Fig. S3. The size distributions of NCs in perovskite NGCs. The grain size of NCs after heat-treatment at 553 K, 573 K, 593 K and 623 K for 1 h. Grain sizes of NCs are much larger than the exciton Bohr diameter of $CsPb(Cl_{1-x}Br_x)_3$ NCs, meaning the regulation of halide composition of NCs lead to the tuning of PL wavelength.

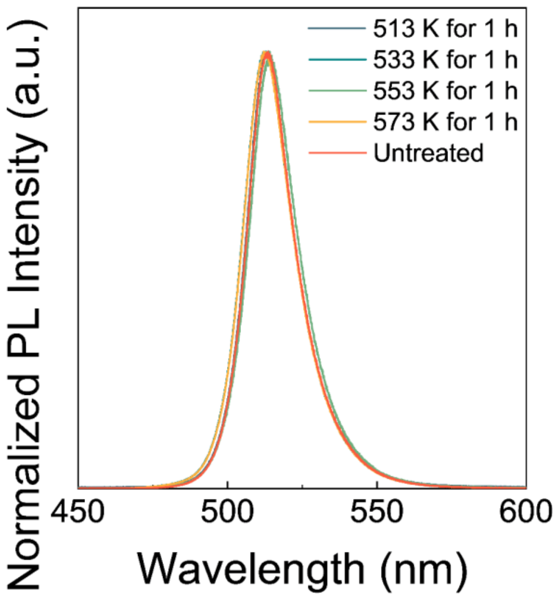

Fig. S4. PL spectra of $CsPbBr_3$ perovskite NGCs under different heat treatment conditions.

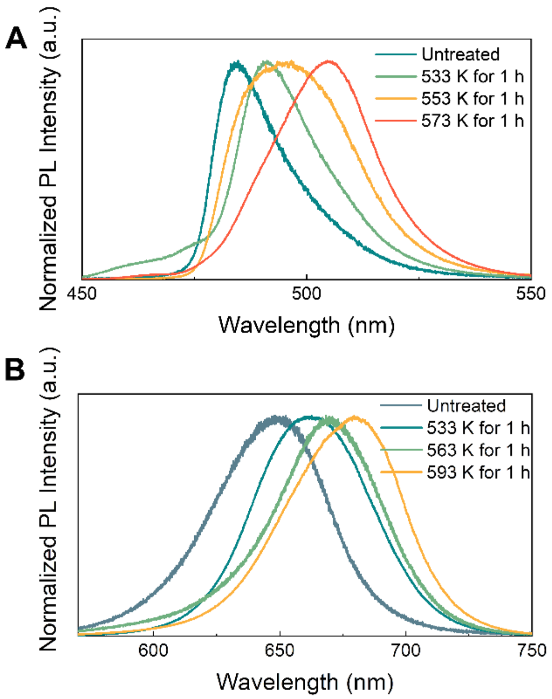

Fig. S5. PL spectra of perovskite NGCs. (**A**) The corresponding PL spectra cover the region from 484 to 505 nm for the NGCs in the Cl–Br doped borate glass(G2) under different heat treatment conditions. (**B**) The corresponding PL spectra cover the region from 648 to 688 nm for the NGCs in Br–I doped borosilicate glass(G4) under different heat treatment conditions.

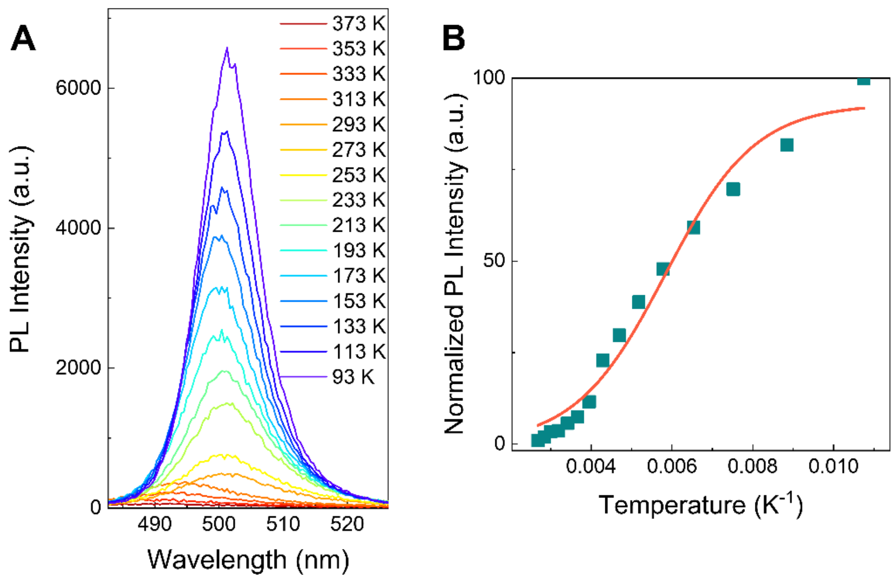

Fig. S6. The temperature-dependent emission spectra. (**A**) PL spectra of perovskite NGCs at the temperature from 93 K to 373 K. (**B**) The fitting result confirmed that the exciton binding energy was 106 meV.

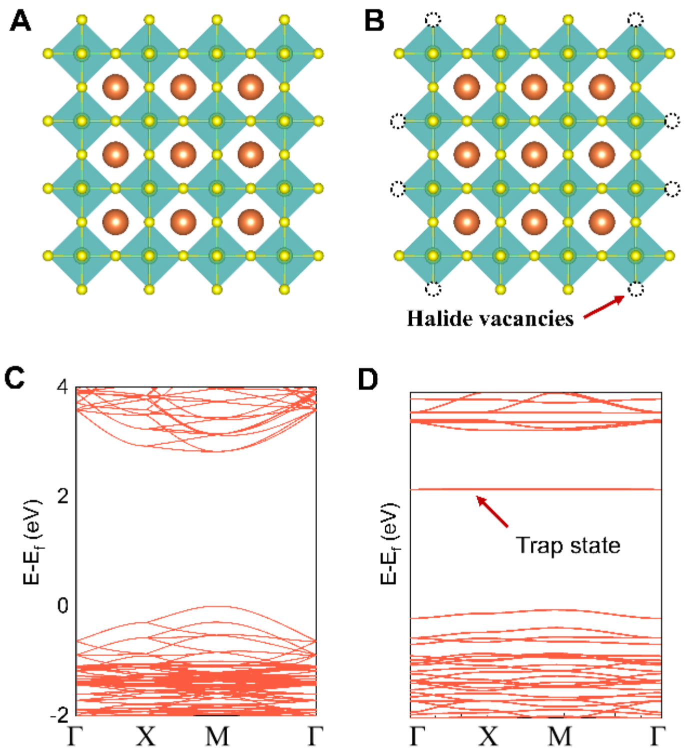

Fig. S7. DFT calculation results of $CsPbBr_3$ supercells. (**A**)-(**B**), The 3a × 3b × 3c supercell structural models of the $CsPbBr_3$ crystal without and with halide vacancy defects, respectively for the subsequent calculations. (**C**) Trap state between the conduction band and valence band of $CsPbBr_3$ PNC without halide vacancy defects and (**D**) with halide vacancy defects calculated by DFT.

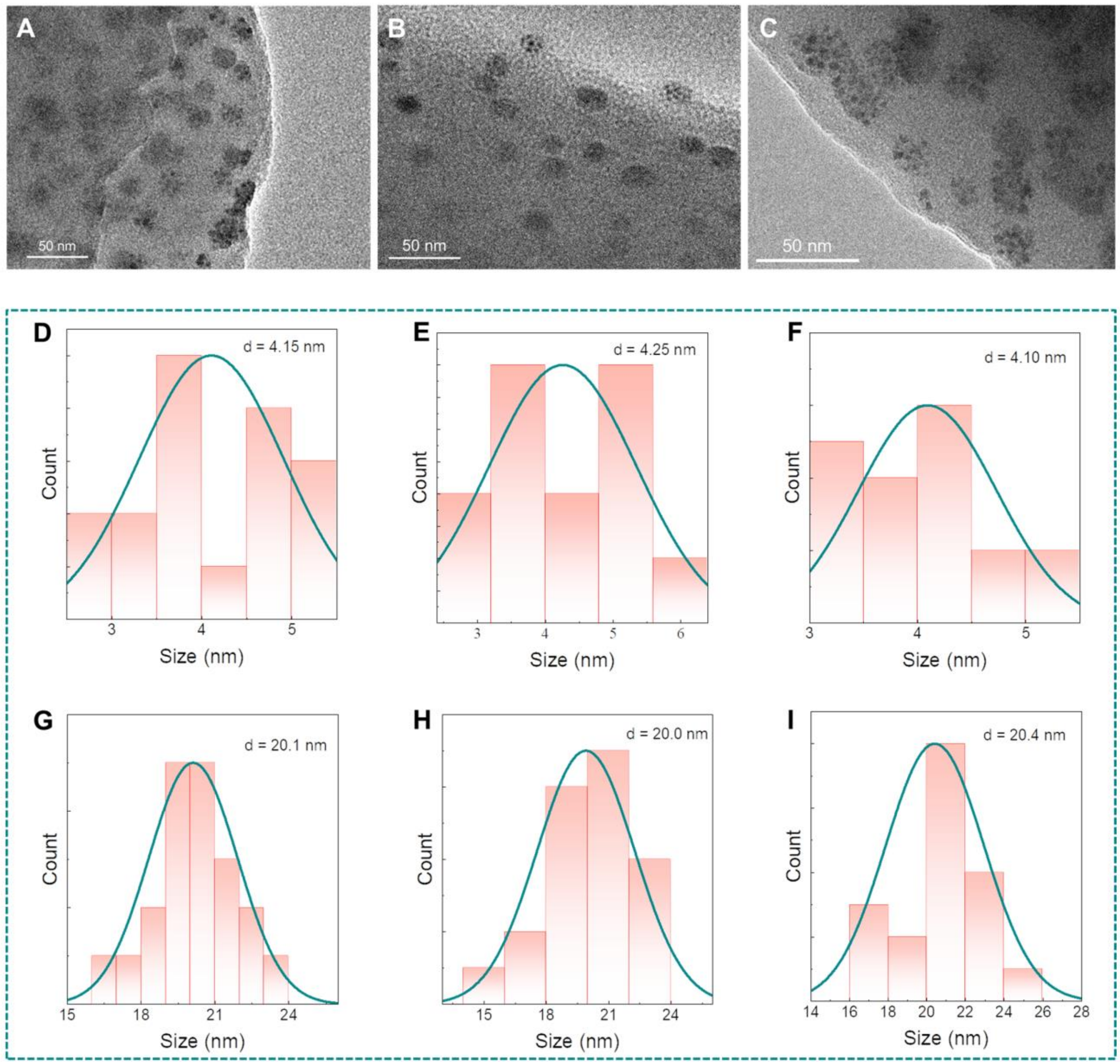

Fig. S8. The TEM images and their size distributions of PNCs. (**A**)-(**C**) TEM images of perovskite NGCs after heat treatment at 693 K for 3 min, 9 min, 13 min and subsequent quenching, respectively. (**D**)-(**F**) The grain size distributions corresponding to (**A**)-(**C**), respectively. (**G**)-(**I**) The size distributions of the amorphous perovskite nanoparticles corresponding to a-c, respectively.

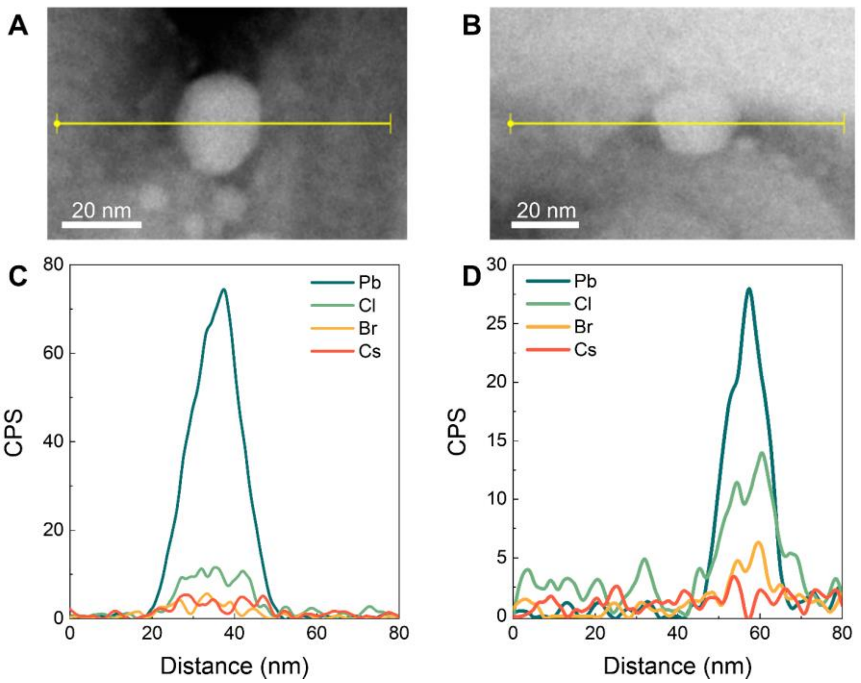

Fig. S9. EDS analysis. (**A**)-(**B**) TEM images of PNCs@amorphous perovskite nanoparticle. (**C**)-(**D**), The corresponding EDS results to (**A**) and (**B**), respectively. The line scan images obtained from EDS analysis reveal the enrichment of perovskite elements, thereby providing further confirmation that the crystals precipitated in the glass are PNCs.

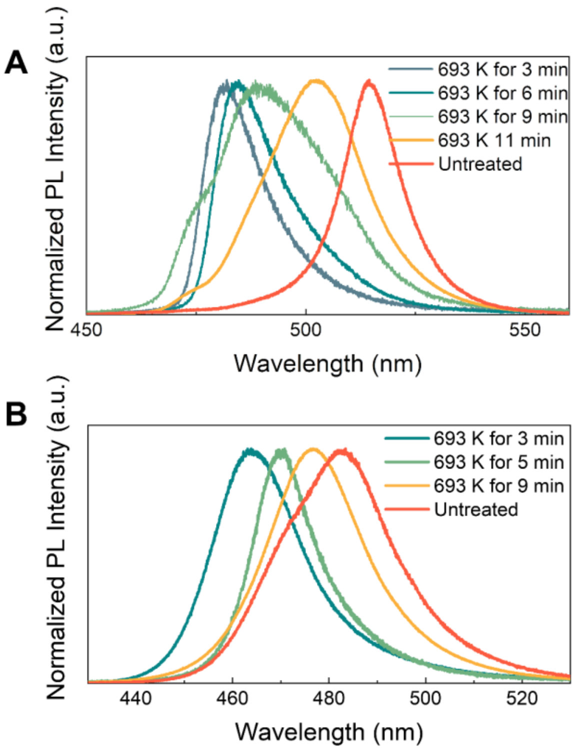

Fig. S10. PL spectra of PNCs-in-glass hierarchical structures. (**A**) The corresponding PL spectra cover the region from 480 to 515 nm in the Cl–Br doped borate glass (G2) under different heat treatment conditions and subsequent quenching. (**B**) The corresponding PL spectra cover the region from 463 to 484 nm in the Cl–Br doped borophosphate glass (G3) under different heat treatment conditions and subsequent quenching.

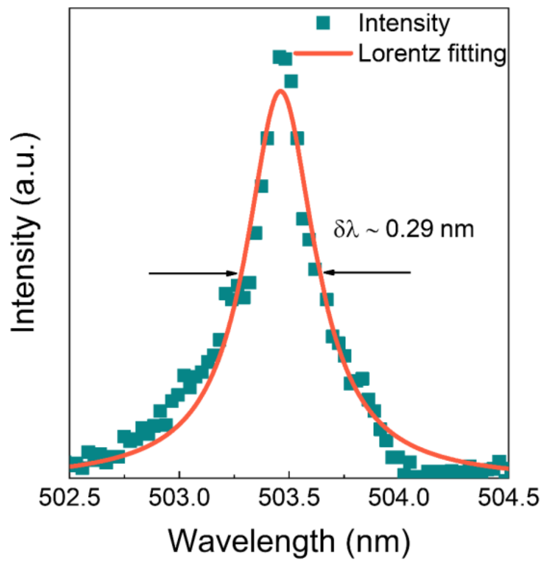

Fig. S11. Enlarged emission spectrum and Lorentz fitting for PNCs-in-glass hierarchical structure.

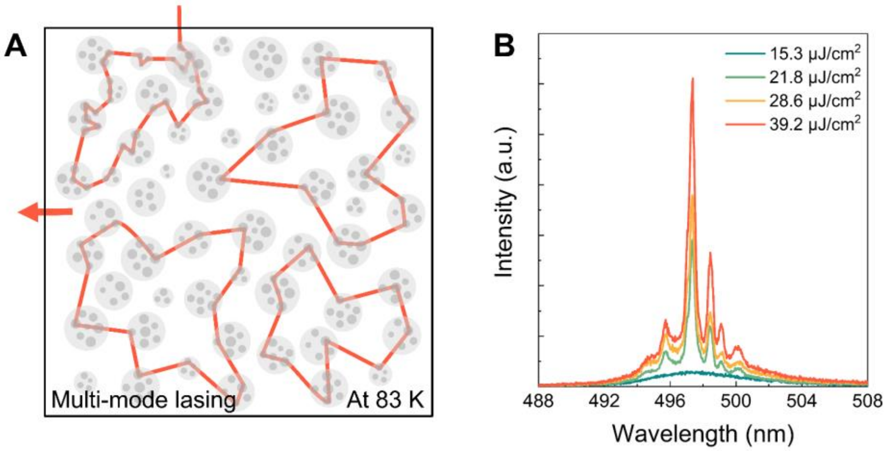

Fig. S12. Multi-mode lasing excited by a 400-nm fs laser. (**A**) The schematic diagram of multiple scattering in the PNCs-in-glass hierarchical structure under a larger excitation spot diameter. (**B**) Power-dependent emission spectra from the PNCs-in-glass hierarchical structure under a larger excitation spot diameter (~ 100 μm).

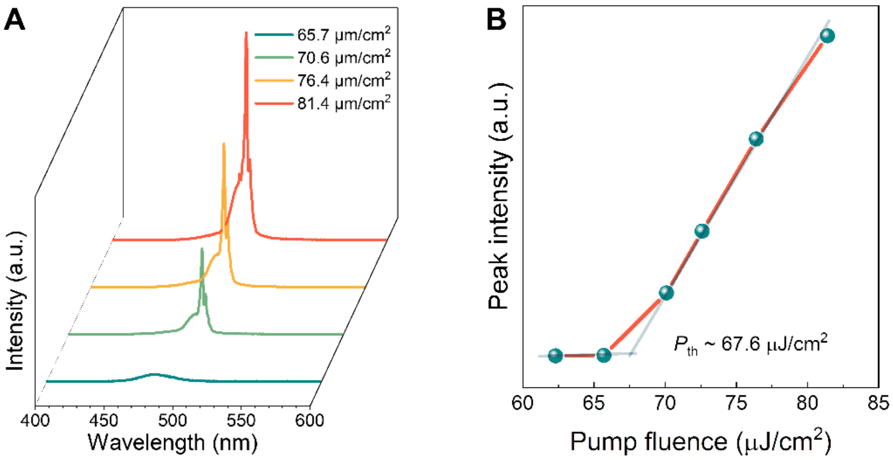

Fig. S13. Random lasing excited by a 400-nm fs laser at 223 K. (**A**) Power-dependent emission spectra from the PNCs-in-glass hierarchical structure. (**B**) Peak intensity and FWHM of the emission spectra as a function of pumping fluence ($P_{th} \sim 67.6$ μJ/cm^2).

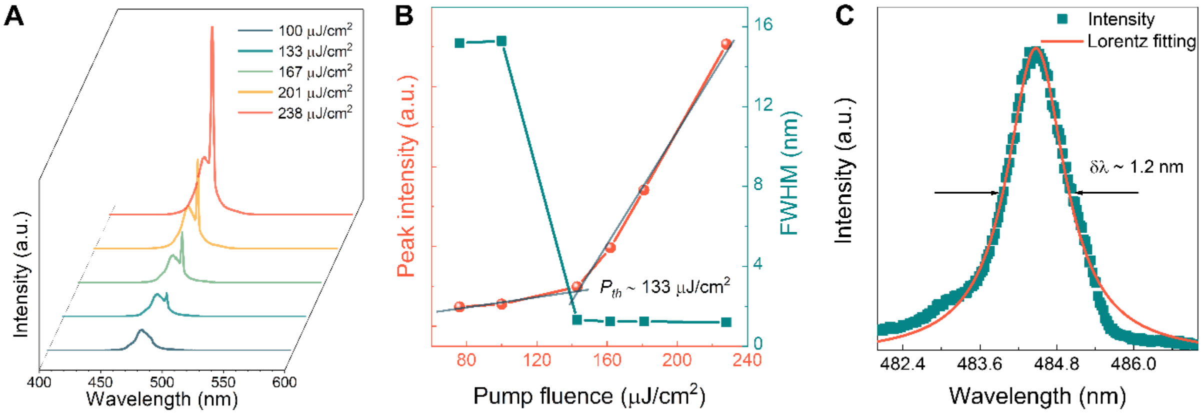

Fig. S14. Single-mode lasing excited by a 400-nm fs laser. (**A**) Power-dependent emission spectra from the PNCs-in-glass hierarchical structure prepared by heat treatment at the temperature of 693 K for 5 min. Narrow emission peaks at ~484 nm are indicative of lasing. (**B**) Peak intensity and FWHM of the emission spectra as a function of pumping fluence (P_{th} ~ 133 μJ/cm^2). (**C**) Enlarged emission spectrum and Lorentz fitting for PNCs-in-glass hierarchical structure.

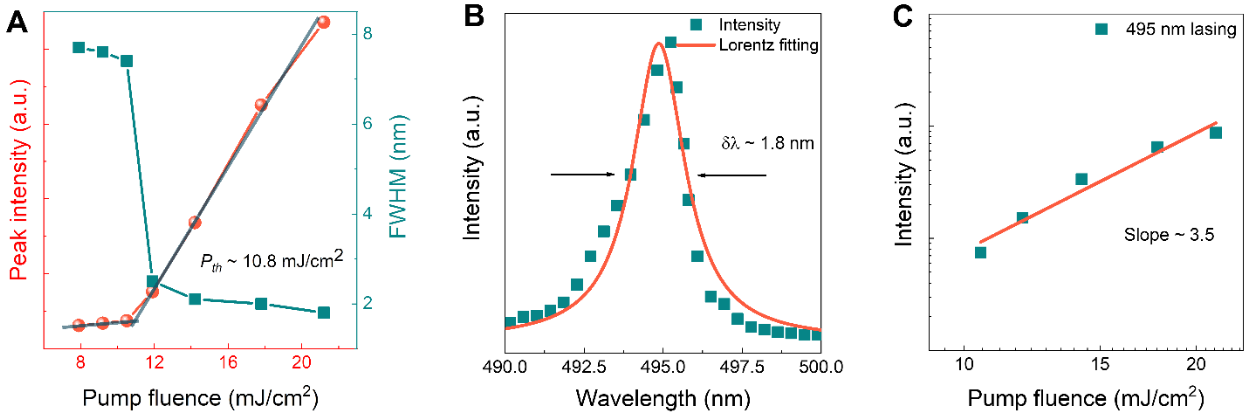

Fig. S15. Single-mode lasing excited by a 400-nm fs laser. (**A**) Power-dependent emission spectra from the PNCs-in-glass hierarchical structure prepared by heat treatment at the temperature of 693 K for 7 min. Narrow emission peaks at ~495 nm are indicative of lasing. (**B**) Peak intensity and FWHM of the emission spectra as a function of pumping fluence (P_{th} ~ 18.7 μJ/cm^2). (**C**) Enlarged emission spectrum and Lorentz fitting for PNCs-in-glass hierarchical structure.

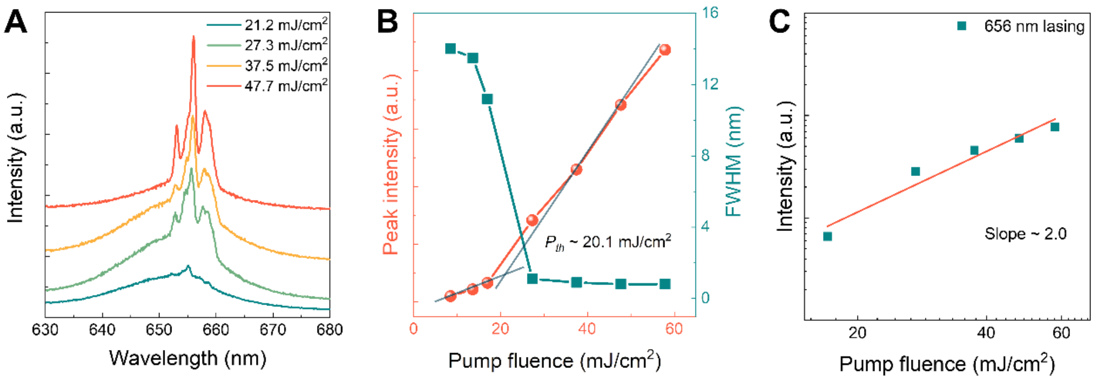

Fig. 16. Random lasing excited by a 1030-nm fs laser. (**A**) Power-dependent emission spectra from the $CsPb(Br_{1-x}I_x)_3$ PNCs-in-glass hierarchical structure prepared by heat treatment at the temperature of 693 K for 5 min. (**B**) Peak intensity and FWHM of the emission spectra as a function of pumping fluence (P_{th} ~ 20.1 mJ/cm^2). (**C**) Lasing intensity as a function of pump fluence.

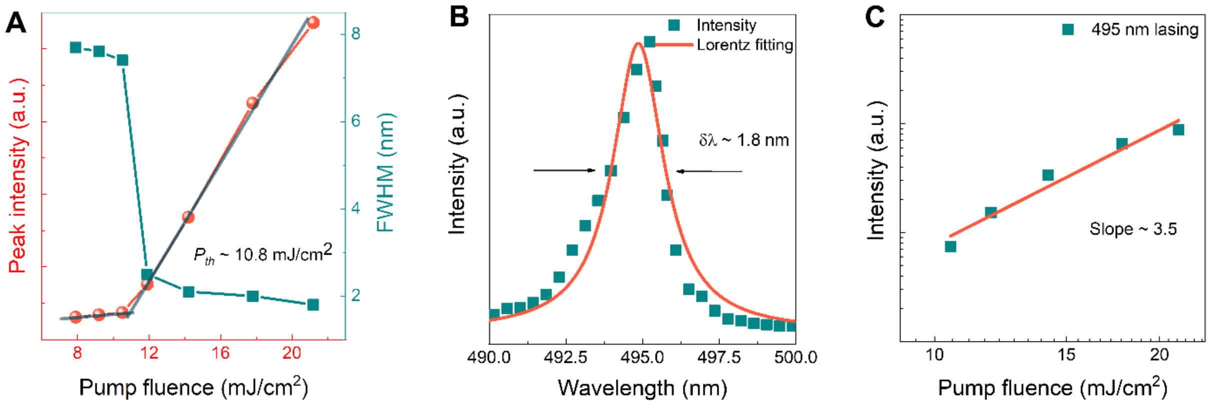

Fig. S17. Single-mode lasing excited by a 1030-nm fs laser. (**A**) Peak intensity and FWHM of the emission spectra as a function of pumping fluence (P_{th} ~ 10.8 mJ/cm^2). (**B**) Enlarged emission spectrum and Lorentz fitting for lasing of the PNCs-in-glass hierarchical structure. (**C**) Lasing intensity as a function of pump fluence.

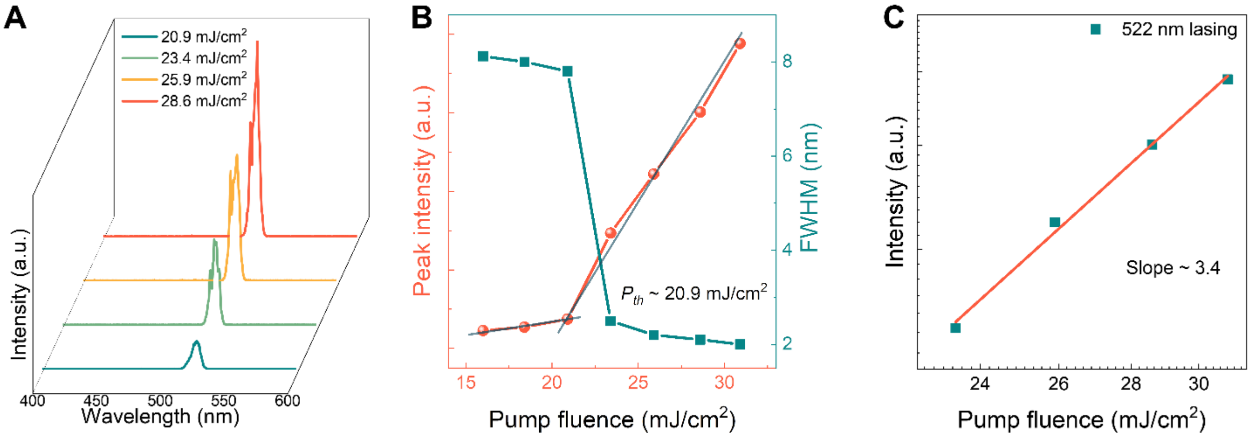

Fig. S18. Random lasing excited by a 1030-nm fs laser at room temperature. (**A**) Power-dependent emission spectra from the PNCs-in-glass hierarchical structure. (**B**) Peak intensity and FWHM of the emission spectra as a function of pumping fluence (P_{th} ~ 20.9 mJ/cm^2). (**C**) Lasing intensity as a function of pump fluence.

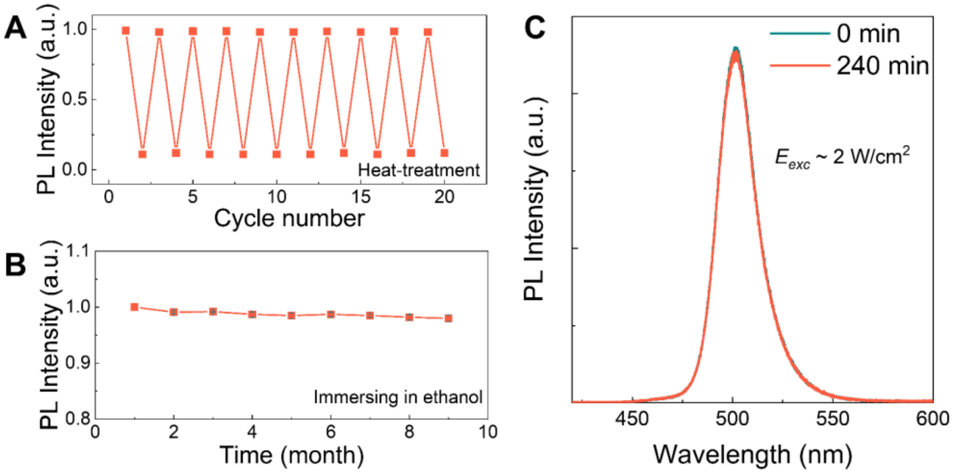

Fig. S19. Stability of PNCs. (**A**) PL intensity of PNCs under heating-cooling (393 K-298 K) cycles. (**B**) PL intensity of PNCs immersed in ethanol for 9 months. (**C**) PL spectra of PNCs illuminated for 240 min by 2 W/cm^2 UV laser.

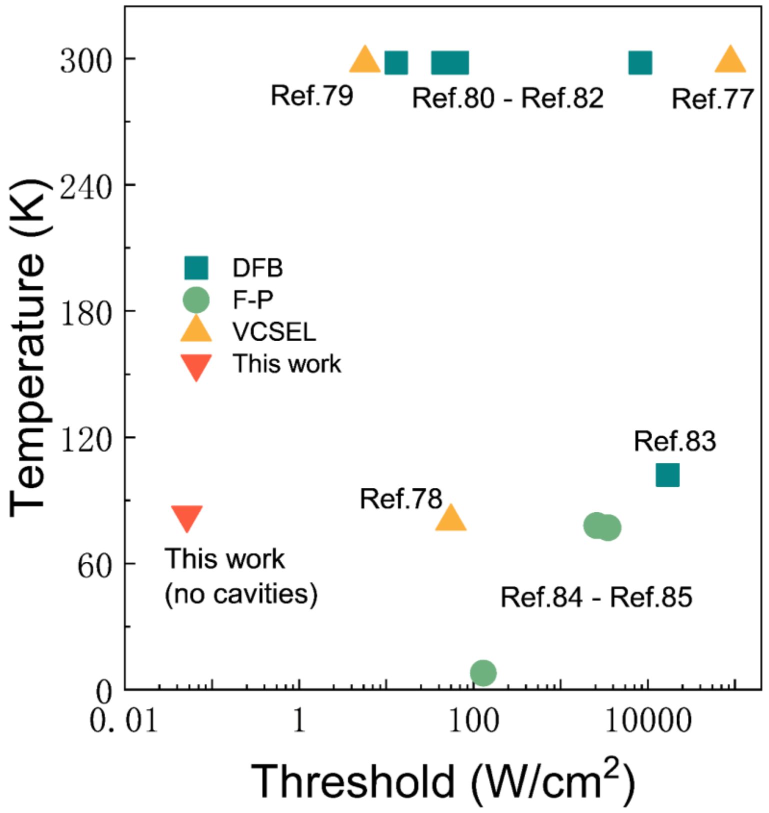

Fig. S20. The threshold of ~ 0.0526 W/cm^2 for the CW lasing with no cavities are significantly lower than those with cavities.

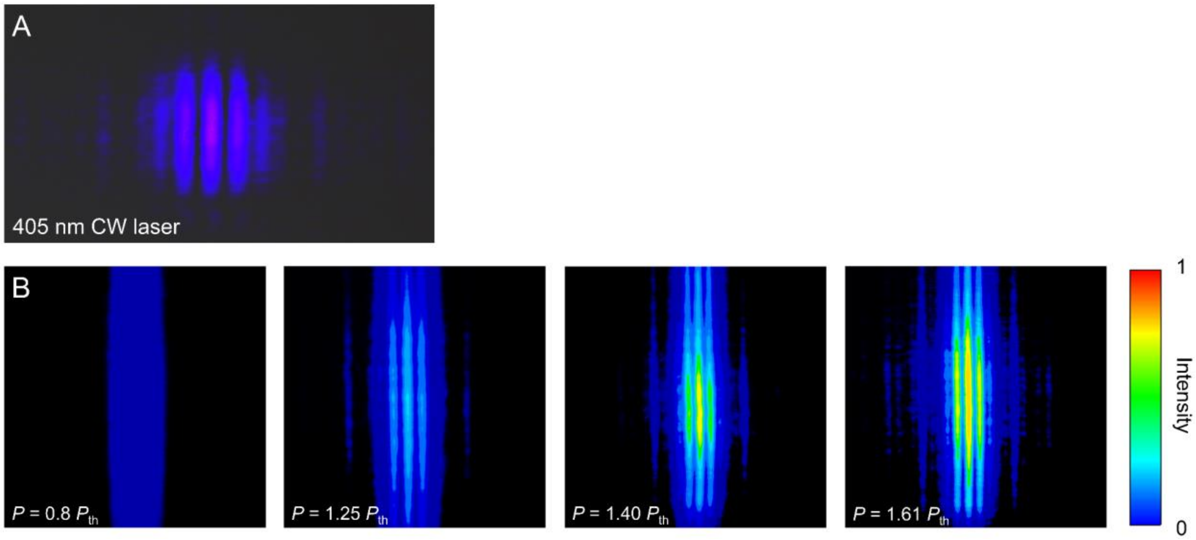

Fig. S21. The interference fringes under different illumination sources. (**A**) Commercial 405 nm CW laser**.** (**B**) Random lasing with the pump fluences ranging from 0.8 P_{th} to 1.61 P_{th}. The Young's double-slit interference setup is used to study the spatial coherence in this work.

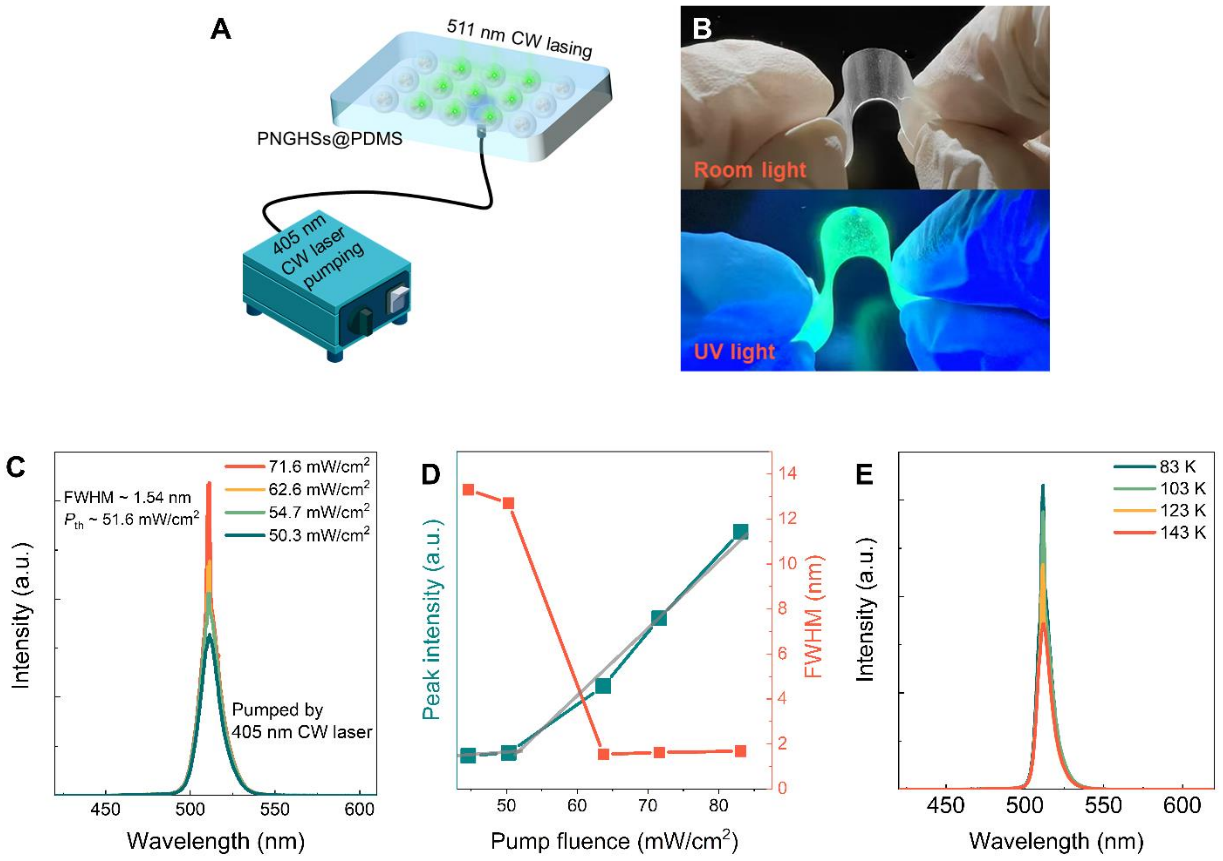

Fig. S22. Flexible random lasing excited by a 405 nm CW laser. (**A**) Schematic illustration of excitation and emission of the flexible PNCs-in-glass@PDMS hierarchical structures. (**B**) Images of the PNCs-in-glass@PDMS hierarchical structures under room light and UV light. (**C**) Power-dependent emission spectra from the PNCs-in-glass@PDMS hierarchical structure at 83 K. (**D**) Peak intensity and FWHM of the emission spectra as a function of pumping fluence. (**E**) Temperature-dependent emission spectra from the PNCs-in-glass@PDMS hierarchical structure.

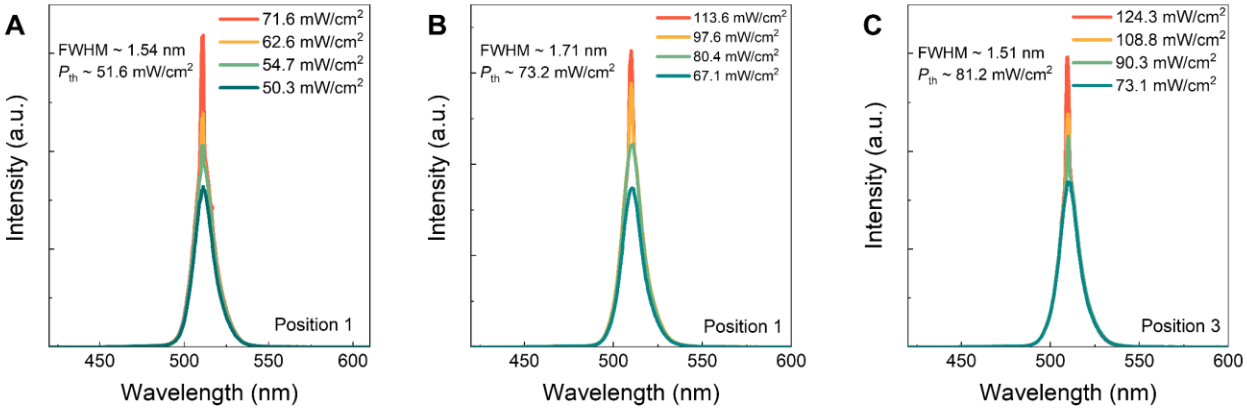

Fig. S23. The emission spectra at different positions from the same hierarchical structure. The observed variations in thresholds are attributed to slight fluctuation in the distribution of PNCs within glass matrix.

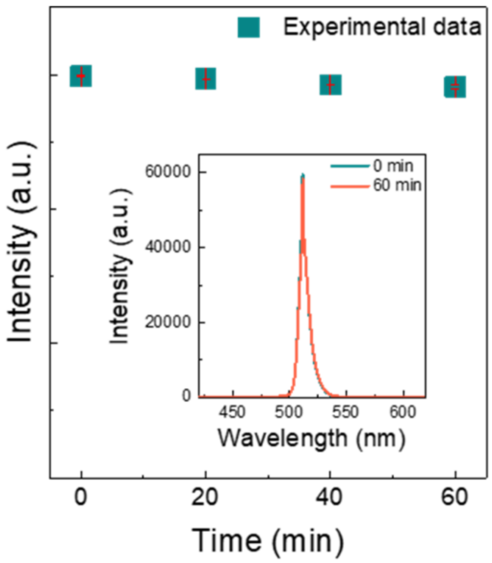

Fig. S24. Peak intensity for the PNCs-in-glass@PDMS hierarchical structure under constant 405 nm CW laser excitation for 60 min at 83 K. The inset shows the emission spectra before and after 405 nm CW laser excitation for 60 min.

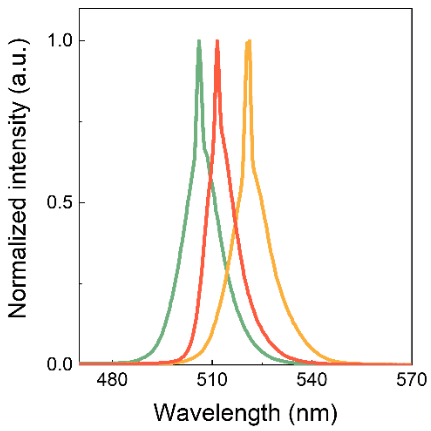

Fig. 25. The emission spectra in the range from 503 nm to 521 nm pumped by a 405 nm laser.

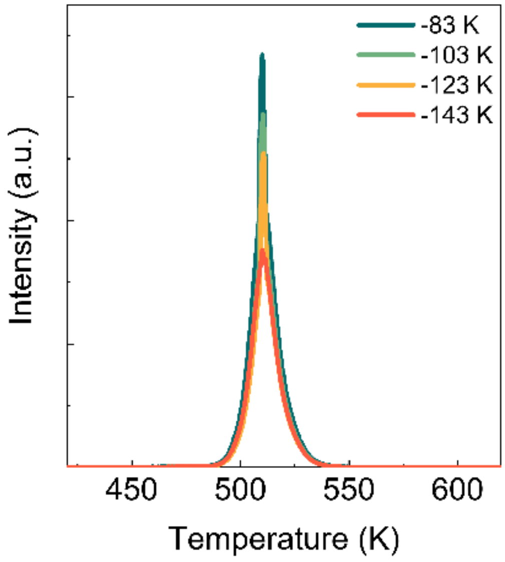

Fig. S26. Emission spectra pumped by a CW laser at various temperatures from the PNCs-in-glass hierarchical structure.

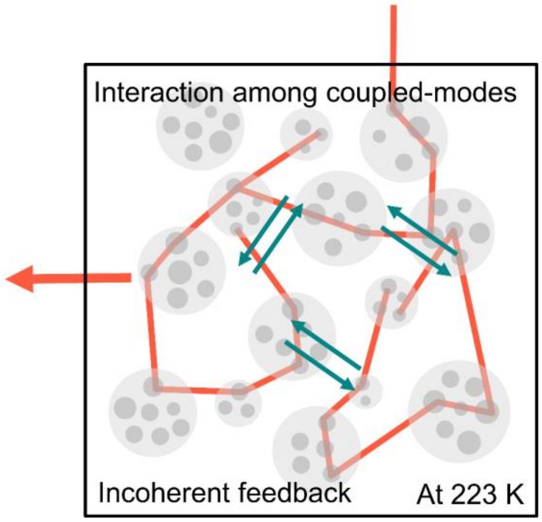

Fig. S27. Mechanism illustration of incoherent feedback. The strong interaction among coupled-modes enables the photos exchange among these modes.

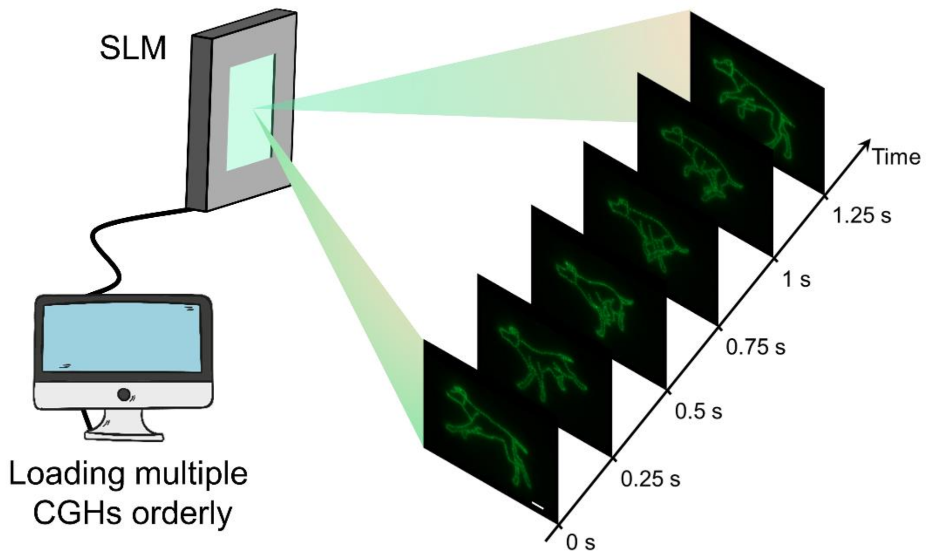

Fig. S28. Demonstration of dynamic holographic display. The images of "running dog" were shown along the time axis (t = 0 s, 0.25 s, 0.5 s … 1.25 s, respectively). The excitation wavelength is 405 nm. The scale bar in Fig. S28 is 20 μm.

Table S1. Comparison for T_g, heat treatment temperatures and heat treatment times.

Crystal	Glass composition (mol%)	Δ*T*(K)	Heat treatment time(h)	Ref.
$CsPbCl_3$ $CsPbBr_3$ $CsPbI_3$	$NH_4H_2PO_4$-Pb_3O_4-Cs_2CO_3-PbX_2-NaX (X = Cl, Br, I)	80-120	2	(*67*)
$Sr_3Al_2O_6$	$SrCO_3$-Al_2O_3	155	5	(*68*)
$BaAl_4O_7$	33$BaCO_3$-67Al_2O_3	91-191	2-24	(*69*)
$Y_3Al_5O_{12}$	77Al_2O_3-23Y_2O_3	63-213	2	(*70*)
$Ca_3Al_2Si_3O_{12}$	$CaCO_3$-Al_2O_3-SiO_2	150-350	2	(*71*)
Eu^{2+}: SrF_2	50SiO_2-20Al_2O_3-20SrF_2-(10-x)NaF-xEuF_3	120-180	2	(*72*)
CdS	80$Pb_2P_2O_7$-10B_2O_3-10ZnO	5	2-5	(*73*)
CdS/ZnS	40SiO_2-28B_2O_3-22Na_2O-10ZnO	52	0.5-5	(*74*)
$CsPbBr_3$	50TeO_2-25WO_3-25LiO_2-3CsBr-6$PbBr_2$	63	1-7	(*75*)
$CsPbBr_3$	40B_2O_3-15P_2O_5-5Na_2O-5K_2O-10ZnO-10Al_2O_3-7Cs_2O-3$PbBr_2$-5NaBr	19-79	3	(*76*)
$CsPb(Cl/Br)_3$	27SiO_2-42B_2O_3-5CaO-10ZnO-5Na_2O-2Cs_2O-1$PbCl_2$-4NaCl-4NaBr	-202	1	**This work**
$CsPb(Cl/Br)_3$	27SiO_2-42B_2O_3-5CaO-10ZnO-5Na_2O-2Cs_2O-1$PbCl_2$-4NaCl-4NaBr	-42	0.05-0.25	**This work**

Notes: $\Delta T = T_x - T_g$, where T_x is the heat treatment temperature, T_g is the glass transition temperature.

Table S2. The suitability of proposed strategy for other glass systems to regulate the PL wavelength of PNCs.

Glass component abbreviation	Crystal	Glass matrix	Glass composition (mol%)	T_g(K)	Heat-treatment conditions	PL range
G1	$CsPb(Cl_{1-x}Br_x)_3$ PNCs	Borosilicate glass	$27SiO_2$-$42B_2O_3$-5CaO-10ZnO-$5Na_2O$-$2Cs_2O$-$1PbCl_2$-4NaCl-4NaBr	735	533-623 K, 1 h	465-515 nm
G2	$CsPb(Cl_{1-x}Br_x)_3$ PNCs	Borate glass	$50B_2O_3$-10CaO-$5K_2O$-10ZnO-$5Cs_2O$-$5PbCl_2$-$5PbBr_2$-5NaCl-5NaBr	723	693 K, 3-13 min	480-515 nm
					533-573 K, 1 h	484-505 nm
G3	$CsPb(Cl_{1-x}Br_x)_3$ PNCs	Borophosphate glass	$10NaPO_3$-$10KPO_3$-$20B_2O_3$-$10Al_2O_3$-10ZnO-$7Cs_2CO_3$-$2PbCl_2$-$1PbBr_2$-10/3NaCl-5/3NaBr-CaF_2	708	693 K, 3-15 min	463-484 nm
G4	$CsPb(Br_{1-x}I_x)_3$ PNCs	Borosilicate glass	$40B_2O_3$-$26SiO_2$-10ZnO-8CaO-$5Na_2CO_3$-$2Cs_2CO_3$-$1PbBr_2$-4NaBr-4NaI	768	693 K, 3-11 min	645-687 nm
					533-593 K, 1 h	648- 680 nm
G5	$CsPbBr_3$ PNCs	Borosilicate glass	$34B_2O_3$-$38SiO_2$-8ZnO-$3SrCO_3$-$8Cs_2CO_3$-$4PbBr_2$-5NaBr-8NaF	746	533-573 K, 1 h	513 nm

Notes: PNCs mean perovskite nanocrystals.

Movie S1. The dynamic holographic display of “a running dog”.

REFERENCES

1. A. Fakharuddin, Perovskite light-emitting diodes. *Nat. Electron.* **5**, 203–216 (2022).

2. Q. Zhang, Q. Shang, R. Su, T. T. H. Do, Q. Xiong, Halide perovskite semiconductor lasers: Materials, cavity design, and low threshold. *Nano Lett.* **21**, 1903–1914 (2021).

3. L. Yi, B. Hou, H. Zhao, X. Liu, X-ray-to-visible light-field detection through pixelated colour conversion. *Nature* **618**, 281–286 (2023).

4. J. J. Yoo, G. Seo, M. R. Chua, T. G. Park, Y. Lu, F. Rotermund, Y.-K. Kim, C. S. Moon, N. J. Jeon, J.-P. Correa-Baena, V. Bulović, S. S. Shin, M. G. Bawendi, J. Seo, Efficient perovskite solar cells via improved carrier management. *Nature* **590**, 587–593 (2021)

5. W. Mao, C. R. Hall, S. Bernardi, Y.-B. Cheng, A. Widmer-Cooper, T. A. Smith, U. Bach, Light-induced reversal of ion segregation in mixed-halide perovskites. *Nat. Mater.* **20**, 55–61 (2021).

6. X.-K. Liu, W. Xu, S. Bai, Y. Jin, J. Wang, R. H. Friend, F. Gao, Metal halide perovskites for light-emitting diodes. *Nat. Mater.* **20**, 10–21 (2021)

7. A. Llordés, G. Garcia, J. Gazquez, D. J. Milliron, Tunable near-infrared and visible-light transmittance in nanocrystal-in-glass composites. *Nature* **500,** 323–326 (2013).

8. K. Sun, D. Tan, X. Fang, X. Xia, D. Lin, J. Song, Y. Lin, Z. Liu, M. Gu, Y. Yue, J. Qiu, Three-dimensional direct lithography of stable perovskite nanocrystals in glass. *Science* **375**, 307–310 (2022).

9. W. Blanc, Y. Gyu Choi, X. Zhang, M. Nalin, K. A. Richardson, G. C. Righini, M. Ferrari, A. Jha, J. Massera, S. Jiang, J. Ballato, L. Petit, The past, present and future of photonic glasses: A review in homage to the united nations international year of glass 2022. *Prog. Mater. Sci.* **134**, 101084 (2023).

10. X. Liu, J. Zhou, S. Zhou, Y. Yue, J. Qiu, Transparent glass-ceramics functionalized by dispersed crystals. *Prog. Mater. Sci.* **97**, 38–96 (2018).

11. V. N. Novikov, A. P. Sokolov, Poisson's ratio and the fragility of glass-forming liquids. *Nature* **431**, 961–963 (2004).

12 J. D. Musgraves, J. Hu, L. Calvez, Eds., *Springer Handbook of Glass* (Springer, 2019).

13. D. S. Wiersma, The physics and applications of random lasers. *Nat. Phys.* **4**, 359–367 (2008).

14. M. Xia, J. Luo, C. Chen, H. Liu, J. Tang, Semiconductor quantum dots-embedded inorganic glasses: Fabrication, luminescent properties, and potential applications. *Adv. Opt. Mater.* **7**, 1900851 (2019).

15. A. Jha, B. Richards, G. Jose, T. Teddy-Fernandez, P. Joshi, X. Jiang, J. Lousteau, Rare-earth ion doped TeO_2 and GeO_2 glasses as laser materials. *Prog. Mater. Sci.* **57**, 1426–1491 (2012).

16. V. I. Klimov, A. A. Mikhailovsky, S. Xu, A. Malko, J. A. Hollingsworth, C. A. Leatherdale, H.-J. Eisler, M. G. Bawendi, Optical gain and stimulated emission in nanocrystal quantum dots. *Science* **290**, 314–317 (2000).

17. B. Redding, M. A. Choma, H. Cao, Speckle-free laser imaging using random laser illumination. *Nat. Photonics* **6**, 355–359 (2012).

18. A. Boschetti, A. Taschin, P. Bartolini, A. K. Tiwari, L. Pattelli, R. Torre, D. S. Wiersma, Spectral super-resolution spectroscopy using a random laser. *Nat. Photonics.* **14**, 177–182 (2020).

19. N. Caselli, A. Consoli, Á. M. Mateos Sánchez, C. López, Networks of mutually coupled random lasers. *Optica* **8**, 193–201 (2021).

20. R. Sapienza, Controlling random lasing action. *Nat. Phys.* **18**, 976–979 (2022).

21. H. Cao, A. P. Mosk, S. Rotter, Shaping the propagation of light in complex media. *Nat. Phys.* **18**, 994–1007 (2022).

22. N. Bachelard, S. Gigan, X. Noblin, P. Sebbah, Adaptive pumping for spectral control of random lasers. *Nat. Phys.* **10**, 426–431 (2014).

23. R. Zheng, J. Ueda, K. Shinozaki, S. Tanabe, Reversible phase segregation and amorphization of mixed-halide perovskite nanocrystals in glass matrices. *J. Phys. Chem. Lett.* **13**, 7809–7815 (2022).

24. L. Protesescu, S. Yakunin, M. I. Bodnarchuk, F. Krieg, R. Caputo, C. H. Hendon, R. X. Yang, A. Walsh, M. V. Kovalenko, Nanocrystals of cesium lead halide perovskites ($CsPbX_3$, X = Cl, Br, and I): Novel optoelectronic materials showing bright emission with wide color gamut. *Nano Lett.* **15**, 3692–3696 (2015).

25. Y. Hu, Y. Ye, W. Zhang, K. Li, Y. Zhou, Y. Zhang, Z. Deng, J. Han, X. Zhao, C. Liu, Laser-induced inverted patterning of nanocrystals embedded glass for micro-light-emitting diodes. *J. Mater. Sci. Technol.* **150**, 138–144 (2023).

26. D. M. Calistru, L. Mihut, S. Lefrant, I. Baltog, Identification of the symmetry of phonon modes in $CsPbCl_3$ in phase IV by Raman and resonance-Raman scattering. *J. Appl. Phys.* **82**, 5391–5395 (1997).

27. H. Xiao, J. Lin, R. Chen, T. Pang, P. Huang, Y. Yu, B. Zhuang, Q. Ye, R. Zhang, D. Chen, Humidity-induced reversible crystallization of laser-printing perovskite quantum dots in glass. *Laser Photonics Rev.* **18**, 2300705 (2024).

28. X. Li, Y. Wu, S. Zhang, B. Cai, Y. Gu, J. Song, H. Zeng, $CsPbX_3$ quantum dots for lighting and displays: Room-temperature synthesis, photoluminescence superiorities, underlying origins and white light-emitting diodes. *Adv. Funct. Mater.* **26**, 2435–2445 (2016).

29. X. Chen, J. Zhou, Z. Xie, Y. Ma, Excitons in confined molecular aggregates. *Inf. Funct. Mater.* **1**, 68–86 (2024).

30. X. Gu, W. Xiang, Q. Tian, S. (Frank) Liu, Rational surface-defect control via designed passivation for high-efficiency inorganic perovskite solar cells. *Angew. Chem. Int. Ed.* **60**, 23164–23170 (2021).

31. M. Hao, S. Cheng, Y. He, W. Xiang, N. Ding, W. Xu, C.-G. Ma, X. Liang, Dy^{3+} doped all-inorganic perovskite nanocrystals glass toward high-performance and high-stability silicon photodetectors. *Laser Photonics Rev.* **2023**, 2200748 (2023).

32. D. Xu, P. Wu, H. Tan, Self-assembled monolayers for perovskite solar cells. *Inf. Funct. Mater.* **1**, 2–25 (2024).

33. J. Zhang, T. Zhang, Z. Ma, F. Yuan, X. Zhou, H. Wang, Z. Liu, J. Qing, H. Chen, X. Li, S. Su, J. Xie, Z. Shi, L. Hou, C. Shan, A multifunctional "halide-equivalent" anion enabling efficient $CsPb(Br/I)_3$ nanocrystals pure-red light-emitting diodes with external quantum efficiency exceeding 23%. *Adv. Mater.* **35**, 2209002 (2022).

34. S. Wang, C. Bi, J. Yuan, L. Zhang, J. Tian, Original core–shell structure of cubic $CsPbBr_3$ @Amorphous $CsPbBr_x$ perovskite quantum dots with a high blue photoluminescence quantum yield of over 80%. *ACS Energy Lett.* **3**, 245–251 (2018).

35. N. M. Lawandy, R. M. Balachandran, A. S. L. Gomes, E. Sauvain, Laser action in strongly scattering media. *Nature* **368**, 436–438 (1994).

36. H. Cao, Random lasers: Development, features and applications. *Opt. Photonics News*, **16**, 24–29 (2005).

37. G.-Q. Liu, X. Feng, N. Wang, Q. Li, R.-B. Liu, Coherent quantum control of nitrogen-vacancy center spins near 1000 kelvin. *Nat. Commun.* **10**, 1344 (2019).

38. H. Cao, J. Y. Xu, Y. Ling, A. L. Burin, E. W. Seeling, X. Liu, R. P. H. Chang, Random lasers with coherent feedback. *IEEE J. Sel. Top. Quantum Electron.* **9**, 111–119 (2003).

39. N. Padiyakkuth, S. Thomas, R. Antoine, N. Kalarikkal, Recent progress and prospects of random lasers using advanced materials. *Mater. Adv.* **3**, 6687–6706 (2022).

40. N. S. Makarov, S. Guo, O. Isaienko, W. Liu, I. Robel, V. I. Klimov, Spectral and dynamical properties of single excitons, biexcitons, and trions in cesium-lead-halide perovskite quantum dots. *Nano Lett.* **16**, 2349–2362 (2016).

41. K. Vynck, R. Pierrat, R. Carminati, L. S. Froufe-Pérez, F. Scheffold, R. Sapienza, S. Vignolini, J. J. Sáenz, Light in correlated disordered media. *Rev. Mod. Phys.* **95**, 045003 (2023).

42. D. S. Wiersma, Random lasers explained? *Nat. Photonics* **3**, 246–248 (2009).

43. Y. Ling, H. Cao, A. L. Burin, M. A. Ratner, X. Liu, R. P. H. Chang, Investigation of random lasers with resonant feedback. *Phys. Rev. A* **64**, 063808 (2001).

44. H. Cao, Y. G. Zhao, S. T. Ho, E. W. Seelig, Q. H. Wang, R. P. H. Chang, Random laser action in semiconductor powder. *Phy. Rev. Lett.* **82**, 2278–2281 (1999).

45. X. Tian, R. Wei, Z. Ma, J. Qiu, Amplified spontaneous emission from perovskite quantum dots inside a transparent glass. *Adv. Opt. Mater.* **10**, 2102483 (2022).

46. F. Wang, H. Zhang, Q. Sun, A. B. Hafsia, Z. Chen, B. Zhang, Y. Xu, W. Jie, Low-temperature solution growth and characterization of halogen (Cl, I)-doped $CsPbBr_3$ crystals. *Cryst. Growth Des.* **20**, 1638–1645 (2020).

47. R. Saran, A. Heuer-Jungemann, A. G. Kanaras, R. J. Curry, Giant bandgap renormalization and exciton-phonon scattering in perovskite nanocrystals. *Adv. Opt. Mater.* **5**, 1700231 (2017).

48. X. Gao, J. Lin, X. Guo, G. He, D. Zou, T. Ishii, D. Zhang, C. Zhao, H. Zhan, J. S. Huang, X. Liu, C. Adachi, C. Qin, L. Wang, Room-temperature continuous-wave microcavity lasers from solution-processed smooth quasi-2D perovskite films with low thresholds. *J. Phys. Chem. Lett.* **14**, 2493–2500 (2023).

49. S. W. Eaton, M. Lai, N. A. Gibson, A. B. Wong, L. Dou, J. Ma, L.-W. Wang, S. R. Leone, P. Yang, Lasing in robust cesium lead halide perovskite nanowires. *Proc. Natl. Acad. Sci. U.S.A.* **113**, 1993–1998 (2016).

50. Y.-S. Park, J. Roh, B. T. Diroll, R. D. Schaller, V. I. Klimov, Colloidal quantum dot lasers. *Nat. Rev. Mater.* **6**, 382–401 (2021).

51. J. Qin, X.-K. Liu, C. Yin, F. Gao, Carrier dynamics and evaluation of lasing actions in halide perovskites. *Trends Chem.* **3**, 34–46 (2021).

52. R. Sapienza, Determining random lasing action. *Nat. Rev. Phys.* **1**, 690–695 (2019).

53. I. D. W. Samuel, E. B. Namdas, G. A. Turnbull, How to recognize lasing. *Nat. Photonics* **3**, 546–549 (2009).

54. C. Zou, X. Cao, Z. Wang, Y. Yang, Y. Lian, B. Zhao, D. Di, Continuous-wave perovskite polariton lasers. *Sci. Adv.* **11**, eadr8826 (2025).

55. J. Song, Q. Shang, X. Deng, Y. Liang, C. Li, X. Liu, Q. Xiong, Q. Zhang, Continuous-wave pumped perovskite lasers with device area below 1 μm2. *Adv. Mater.* **35**, e2302170 (2023).

56. W. Z. W. Ismail, D. Liu, S. Clement, D. W. Coutts, E. M. Goldys, J. M. Dawes, Spectral and coherence signatures of threshold in random lasers. *J. Opt.* **16**, 105008 (2014).

57. F. Zhao, A. Ren, P. Li, Y. Li, J. Wu, Z. M. Wang, Toward continuous-wave pumped metal halide perovskite lasers: Strategies and challenges. *ACS Nano* **16**, 7116–7143 (2022).

58. S. Lv, D. Wang, J. Tang, Z. Liu, H. Inoue, B. Tang, Z. Sun, L. Wondraczek, J. Qiu, S. Zhou, Transparent composites for efficient neutron detection. *Nat. Commun.* **15**, 6746 (2024).

59. M. Wang, P. Zhang, M. Shamsi, J. L. Thelen, W. Qian, V. K. Truong, J. Ma, J. Hu, M. D. Dickey, Tough and stretchable ionogels by in situ phase separation. *Nat. Mater.* **21**, 359–365 (2022).

60. F. Chen, X. Li, Y. Yu, Q. Li, H. Lin, L. Xu, H. C. Shum, Phase-separation facilitated one-step fabrication of multiscale heterogeneous two-aqueous-phase gel. *Nat. Commun.* **14**, 2793 (2023).

61. S. E. Neumann, J. Kwon, C. Gropp, L. Ma, R. Giovine, T. Ma, N. Hanikel, K. Wang, T. Chen, S. Jagani, R. O. Ritchie, T. Xu, O. M. Yaghi, The propensity for covalent organic frameworks to template polymer entanglement. *Science* **383**, 1337–1343 (2024).

62. Q. Zhang, S. Dong, P. Shao, Y. Zhu, Z. Mu, D. Sheng, T. Zhang, X. Jiang, R. Shao, Z. Ren, J. Xie, X. Feng, B. Wang, Covalent organic framework–based porous ionomers for high-performance fuel cells. *Science* **378**, 181–186 (2022).

63. Y. Hou, Z. Zhou, C. Zhang, J. Tang, Y. Fan, F.-F. Xu, Y. S. Zhao, Full-color flexible laser displays based on random laser arrays. *Sci. China Mater.* **64**, 2805–2812 (2021).

64. V. I. Klimov, Optical nonlinearities and ultrafast carrier dynamics in semiconductor nanocrystals. *J. Phys. Chem. B* **104**, 6112–6123 (2000).

65. M. R. Luo Eds., *Encyclopedia of Color Science and Technology* (Springer, 2016).

66. V. I. Klimov, J. A. McGuire, R. D. Schaller, V. I. Rupasov, Scaling of multiexciton lifetimes in semiconductor nanocrystals. *Phys. Rev. B* **77**, 195324 (2008).

67. P. Li, W. Xie, W. Mao, Y. Tian, F. Huang, S. Xu, J. Zhang, A new whole family perovskites quantum dots ($CsPbX_3$, X=Cl, Br, I) phosphate glasses with full spectral emissions. *J. Alloys Compd.* **817**, 153338 (2020).

68. S. Alahraché, K. Al Saghir, S. Chenu, E. Véron, D. De Sousa Meneses, A. I. Becerro, M. Ocaña, F. Moretti, G. Patton, C. Dujardin, F. Cussó, J.-P. Guin, M. Nivard, J.-C. Sangleboeuf, G. Matzen, M. Allix, Perfectly transparent $Sr_3Al_2O_6$ polycrystalline ceramic elaborated from glass crystallization. *Chem. Mater.* **25**, 4017–4024 (2013).

69. M. Allix, S. Alahrache, F. Fayon, M. Suchomel, F. Porcher, T. Cardinal, G. Matzen, Highly transparent $BaAl_4O_7$ polycrystalline ceramic obtained by full crystallization from glass. *Adv. Mater.* **24**, 5570–5575 (2012).

70. X. Ma, X. Li, J. Li, C. Genevois, B. Ma, A. Etienne, C. Wan, E. Véron, Z. Peng, M. Allix, Pressureless glass crystallization of transparent yttrium aluminum garnet-based nanoceramics. *Nat. Commun.* **9**, 1175 (2018).

71. T. Irifune, K. Kawakami, T. Arimoto, H. Ohfuji, T. Kunimoto, T. Shinmei, Pressure-induced nano-crystallization of silicate garnets from glass. *Nat. Commun.* **7**, 13753 (2016).

72. Q. Luo, X. Qiao, X. Fan, X. Zhang, Luminescence properties of Eu^{2+}-doped glass ceramics containing SrF_2 nanocrystals. *J. Am. Ceram. Soc.* **93**, 2684–2688 (2010).

73. G. A. Dos Santos, R. G. Capelo, C. Liu, D. Manzani, In-situ synthesis of luminescent CdS quantum dots embedded in phosphate glass. *J. Non Cryst. Solids* **587**, 121599 (2022).

74. S. Y. Janbandhu, S. Ct, S. R. Munishwar, J. R. Jayaramaiah, R. S. Gedam, Borosilicate glasses containing CdS/ZnS QDs: A heterostructured composite with enhanced degradation of IC dye under visible-light. *Chemosphere* **286**, 131672 (2022).

75. E. Erol, O. Kıbrıslı, M. Çelikbilek Ersundu, A. E. Ersundu, Size-controlled emission of long-time durable $CsPbBr_3$ perovskite quantum dots embedded tellurite glass nanocomposites. *Chem. Eng. J.* **401**, 126053 (2020).

76. Y. Du, X. Wang, D. Shen, J. Yuan, Y. Wang, S. Yan, S. Han, Y. Tao, D. Chen, Precipitation of $CsPbBr_3$ quantum dots in borophosphate glasses inducted by heat-treatment and UV-NIR ultrafast lasers. *Chem. Eng. J.* **401**, 126132 (2020).

77. M. S. Alias, Z. Liu, A. Al-atawi, T. K. Ng, T. Wu, B. S. Ooi, Continuous-wave optically pumped green perovskite vertical-cavity surface-emitter. *Opt. Lett.* **42**, 3618–3621 (2017).

78. H. Zhang, C. Zou, Y. Chen, L. Wu, W. Wen, B. Du, S. Feng, J. Shang, C. Cong, T. Yu, Continuous-wave vertical cavity surface-emitting lasers based on single crystalline lead halide perovskites. *Adv. Opt. Mater.* **9**, 2001982 (2021).

79. H. Zhang, Y. Hu, W. Wen, B. Du, L. Wu, Y. Chen, S. Feng, C. Zou, J. Shang, H. J. Fan, T. Yu, Room-temperature continuous-wave vertical-cavity surface-emitting lasers based on 2D layered organic–inorganic hybrid perovskites. *APL Mater.* **9**, 071106 (2021).

80. F. Fan, O. Voznyy, R. P. Sabatini, K. T. Bicanic, M. M. Adachi, J. R. McBride, K. R. Reid, Y.-S. Park, X. Li, A. Jain, R. Quintero-Bermudez, M. Saravanapavanantham, M. Liu, M. Korkusinski, P. Hawrylak, V. I. Klimov, S. J. Rosenthal, S. Hoogland, E. H. Sargent, Continuous-wave lasing in colloidal quantum dot solids enabled by facet-selective epitaxy. *Nature* **544**, 75–79 (2017).

81. C. Qin, A. S. D. Sandanayaka, C. Zhao, T. Matsushima, D. Zhang, T. Fujihara, C. Adachi, Stable room-temperature continuous-wave lasing in quasi-2D perovskite films. *Nature* **585**, 53–57 (2020).

82. Z. Li, J. Moon, A. Gharajeh, R. Haroldson, R. Hawkins, W. Hu, A. Zakhidov, Q. Gu, Room-temperature continuous-wave operation of organometal halide perovskite lasers. *ACS Nano* **12**, 10968–10976 (2018).

83. Y. Jia, R. A. Kerner, A. J. Grede, B. P. Rand, N. C. Giebink, Continuous-wave lasing in an organic–inorganic lead halide perovskite semiconductor. *Nat. Photonics* **11**, 784–788 (2017).

84. T. J. S. Evans, A. Schlaus, Y. Fu, X. Zhong, T. L. Atallah, M. S. Spencer, L. E. Brus, S. Jin, X.-Y. Zhu, Continuous-wave lasing in cesium lead bromide perovskite nanowires. *Adv. Opt. Mater.* **6**, 1700982 (2018).

85. Q. Shang, M. Li, L. Zhao, D. Chen, S. Zhang, S. Chen, P. Gao, C. Shen, J. Xing, G. Xing, B. Shen, X. Liu, Q. Zhang, Role of the exciton–polariton in a continuous-wave optically pumped $CsPbBr_3$ perovskite laser. *Nano Lett.* **20**, 6636–6643 (2020).
